# Role of estrogen receptors in health and disease

**DOI:** 10.3389/fendo.2022.839005

**Published:** 2022-08-18

**Authors:** Peng Chen, Bo Li, Ling Ou-Yang

**Affiliations:** Department of Obstetrics and Gynecology, Shengjing Hospital of China Medical University, Shenyang, China

**Keywords:** estrogen receptor alpha, estrogen receptor beta, signaling pathway, mediation, G-protein-coupled estrogen receptor 1

## Abstract

Estrogen receptors (ERs) regulate multiple complex physiological processes in humans. Abnormal ER signaling may result in various disorders, including reproductive system-related disorders (endometriosis, and breast, ovarian, and prostate cancer), bone-related abnormalities, lung cancer, cardiovascular disease, gastrointestinal disease, urogenital tract disease, neurodegenerative disorders, and cutaneous melanoma. ER alpha (ERα), ER beta (ERβ), and novel G-protein-coupled estrogen receptor 1 (GPER1) have been identified as the most prominent ERs. This review provides an overview of ERα, ERβ, and GPER1, as well as their functions in health and disease. Furthermore, the potential clinical applications and challenges are discussed.

## Introduction

Estrogen, a steroid compound, is primarily produced by the ovaries and placenta in females (1). The adrenal cortex of males also produce estrogen. Estrogens play important modulatory roles in physiological and pathophysiological processes (2). They perform their roles mainly by interacting with estrogen receptors (ERs). ERs are ligand-dependent transcription factors that regulate gene transcription through estrogen response elements (EREs), thereby facilitating the normal biological functions of estrogens. However, abnormal ER signaling can result in multiple disorders, including various cancers (2) and gynecological disorders, such as polycystic ovary syndrome and endometriosis (3). Three types of ERs, classical alpha (ERα) and beta (ERβ), as well as non-classical G protein-coupled estrogen receptor 1 (GPER1), are involved in several biological processes. In this review, we systematically illustrate the essential roles of these ERs in mediating various physiological and pathological processes.

## Estrogens and ERs

### Estrogens

Estrogen, a lipid-soluble steroid hormone, is one of the most important female sex hormones. It is predominantly produced by the ovaries, testes, and adrenal cortex, and performs various crucial physiological functions. Estrogens are divided into two major categories, endogenous and exogenous, based on their origin. Endogenous estrogens are secreted by glands or cells in the body of living organisms, including estrogen in animals and phytoestrogens (such as genistein and zearalenone) in plants (4). In contrast, exogenous estrogens are derived from synthetic estrogens, food, and drugs (5).

To date, four estrogens, estrone (E1), 17β-estradiol (E2), estriol (E3), and estetrol (E4), have been identified in humans (6). The term “estrogen” typically refers to E2 owing to its widespread distribution and active physiological functions in multiple tissues and organ systems (1). E2 plays a major role in developing secondary female sex characteristics, regulates the menstrual cycle, and growth of the endometrial lining from menarche to menopause (6). E2 also demonstrates a high affinity for ERα, ERβ, and GPER1 (7). E1 is a weaker form of estrogen owing to its lower binding affinity for ERα and ERβ and is the predominant estrogen in women undergoing menopause. E3 is the primary estrogen secreted by the placenta during pregnancy. Consequentially, E3 levels are negligible in non-pregnant women or men compared to their manifold increase during pregnancy (6). Compared to E2, E3 has a lower affinity for ERα (14%) and ERβ (21%) (6). Similar to E3, E4 is a natural fetal estrogen that is detectable only during pregnancy. E4 possesses an overall lower affinity for ERs than E2 (6). All four estrogens consist of 18 carbon atoms with similar chemical functions and structures.

The most common exogenous estrogens contain those metabolized and excreted by living organisms into the environment, selective estrogen receptor modulators (SERMs) targeting the ER for the treatment of endocrine disorders (such as diethylstilbestrol and raloxifene), and environmental pollutants generated by industrial and agricultural activities (such as polycyclic aromatic hydrocarbons and phenolic compounds) (8, 9). The affinity of these exogenous estrogens to the native receptors is relatively low (micromolar values), and their structure is different from that of natural estrogens (10).

Estrogens additionally play crucial roles in regulating the cardiovascular system, liver, pancreas, bone, brain, and immune system (2). Estrogens also participate in regulating spermatogenesis and male fertility (11). Notably, the physiological functions regulated by estrogens are mediated by ERs.

### Discovery of ERs

Elwood Jensen first established the existence of ERs in 1958 by demonstrating that female reproductive tissue can take up estrogen by binding to proteins (1), suggesting that estrogen-bond receptors can stimulate gene transcription after migrating to the nucleus (12). In 1985, the first human ER, termed ERα, was cloned (13). Subsequently, ERβ (or ERβ1) was discovered by Kuiper et al. in 1996 (14). ERα and ERβ are highly homologous nuclear ERs (nERs), isolated using traditional biochemical approaches.

In 2012, a new G protein-coupled membrane receptor (mER), GPER1, was identified using molecular cloning techniques (15). The history of GPER1 stemmed from 1997, when a 7-transmembrane receptor named GPR30, was identified and cloned (16). Some years later, E2 were demonstrated to induce rapidly cell cascades through GPR30 in breast cancer cells lacking ERs, but expressing GPR30 (17, 18). Interestingly, these rapidly responses were blocked when GPR30 silenced (18). Next, experimental data indicated a direct bind to GPR30 by E2, suggesting that it may work as a membrane-bound ER (19, 20). Subsequently, GPR30 was officially renamed G protein-coupled estrogen receptor 1 (also known as GPER or GPER1) in 2007 (21, 22).

### Structure of ERs

**Figure 1:**
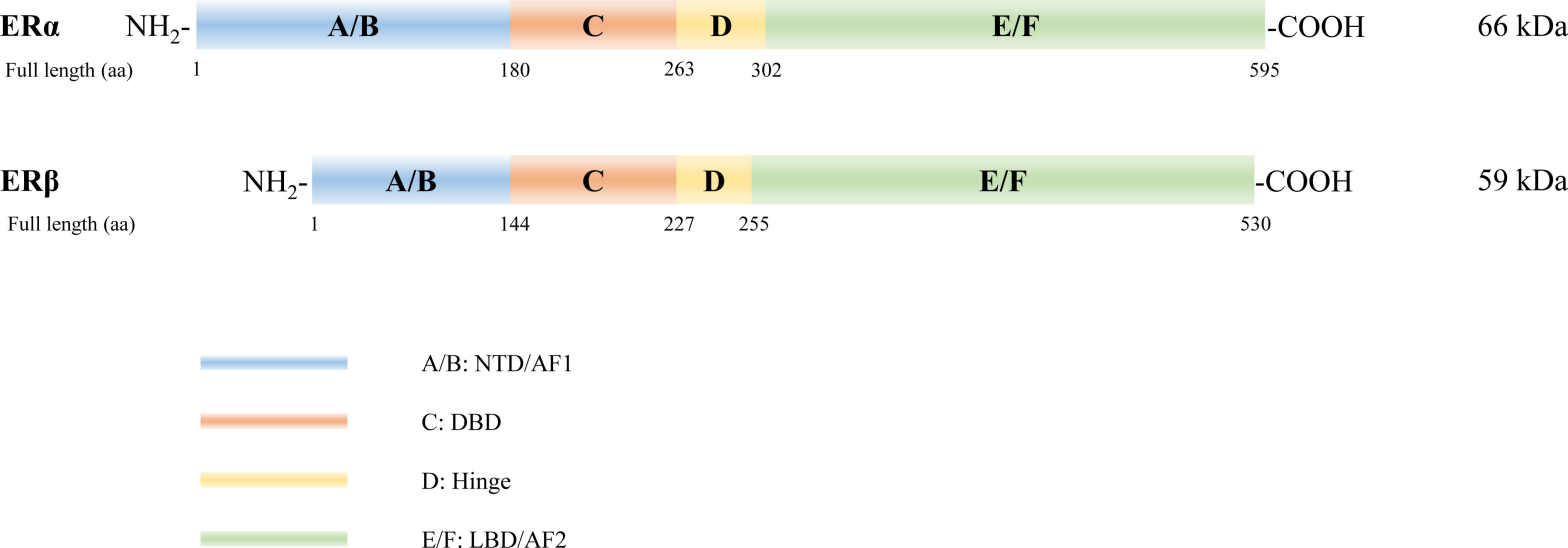
Structure of estrogen receptors (ERs). Six structural and functional domains are highlighted: A, B domain (amino-terminal or NH_2_-terminal domain [NTD], activation function 1 [AF1]); C domain (DNA binding domain [DBD]); D domain (hinge region connecting the C and E domain); E, F domain (carboxyl- or COOH-terminal, ligand-binding domain [LBD], AF2).

The ER protein molecule consists of A/B, C, D, and E/F domains, from amino to carboxyl terminals (**Figure 1**). ERα and ERβ are encoded by *ESR1* on chromosome 6 (6q25.1) (23) and *ESR2* on chromosome 14 (14q23.2) (24), respectively. Full-length ERα and ERβ are composed of 595 and 530 amino acids with relative molecular masses of 66 and 59 kDa, respectively. The A/B domain, which is the amino- or N-terminal domain (NTD), participates in the transcriptional activation of target genes and is associated with receptor specificity. The C domain, known as the DNA-binding domain (DBD), is highly conserved and enhances the DNA-binding ability of ERs. Domain D, a hinge region connecting the C and E domains, contains a nuclear localization signal that binds to heat shock proteins and stabilizes the DNA-binding function of the C domain. The E/F domain located in the carboxyl-terminal, also known as the ligand-binding domain (LBD), displays a complex regulatory function. Both ERα and ERβ exhibit their activation function through activation function 1 (AF1) and AF2 located in the NTD land LBD, respectively, and mediate synergistic transcriptional regulation (25). Notably, ERα and ERβ demonstrate 16%, 97%, and 59% similarity in their NTD, DBD, and LBD, respectively (26).

**Figure 2:**
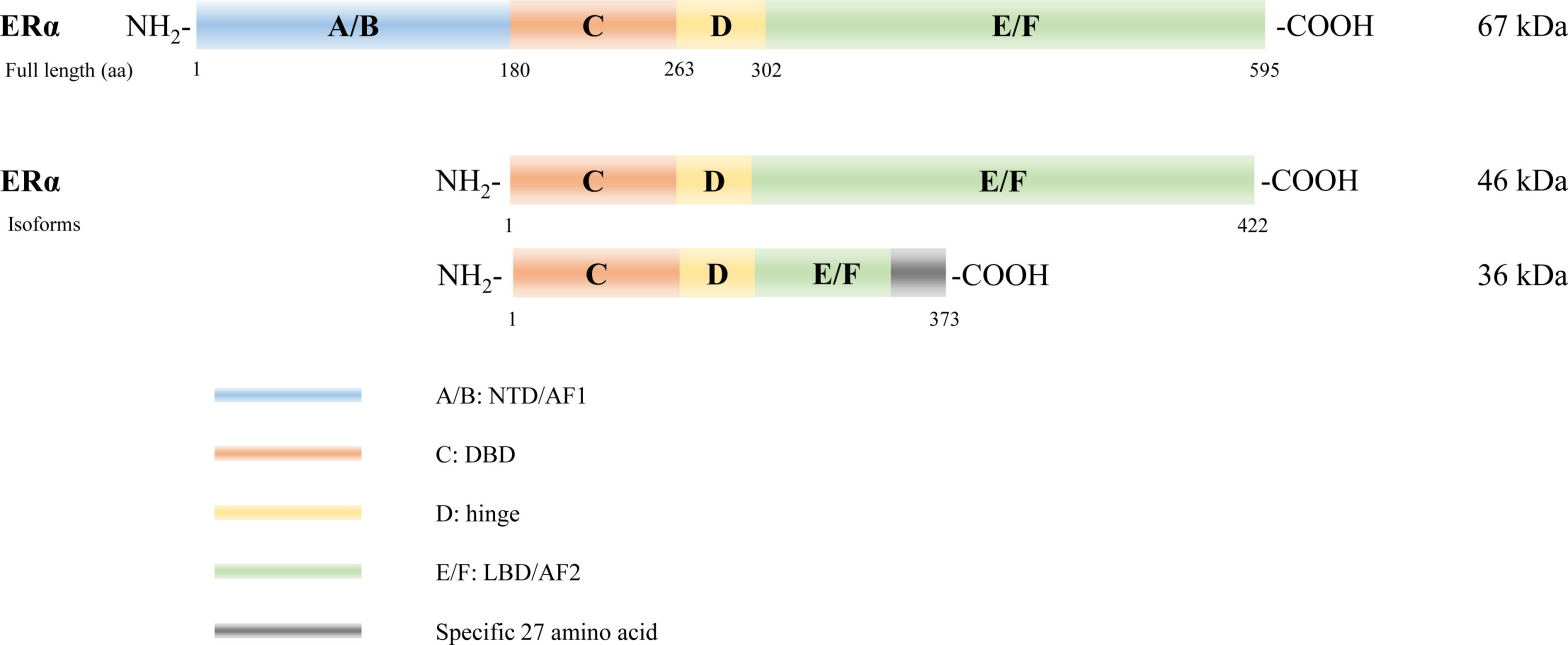
Estrogen receptor alpha (ERα) isoforms. Six structural and functional domains are highlighted: A, B domain (amino- or NH_2_-terminal domain [NTD], AF1), C domain (DNA binding domain [DBD]), D domain (hinge region connecting the C and E domain), E, F domain (carboxyl- or COOH-terminal, ligand-binding domain [LBD], AF2).

**Figure 3:**
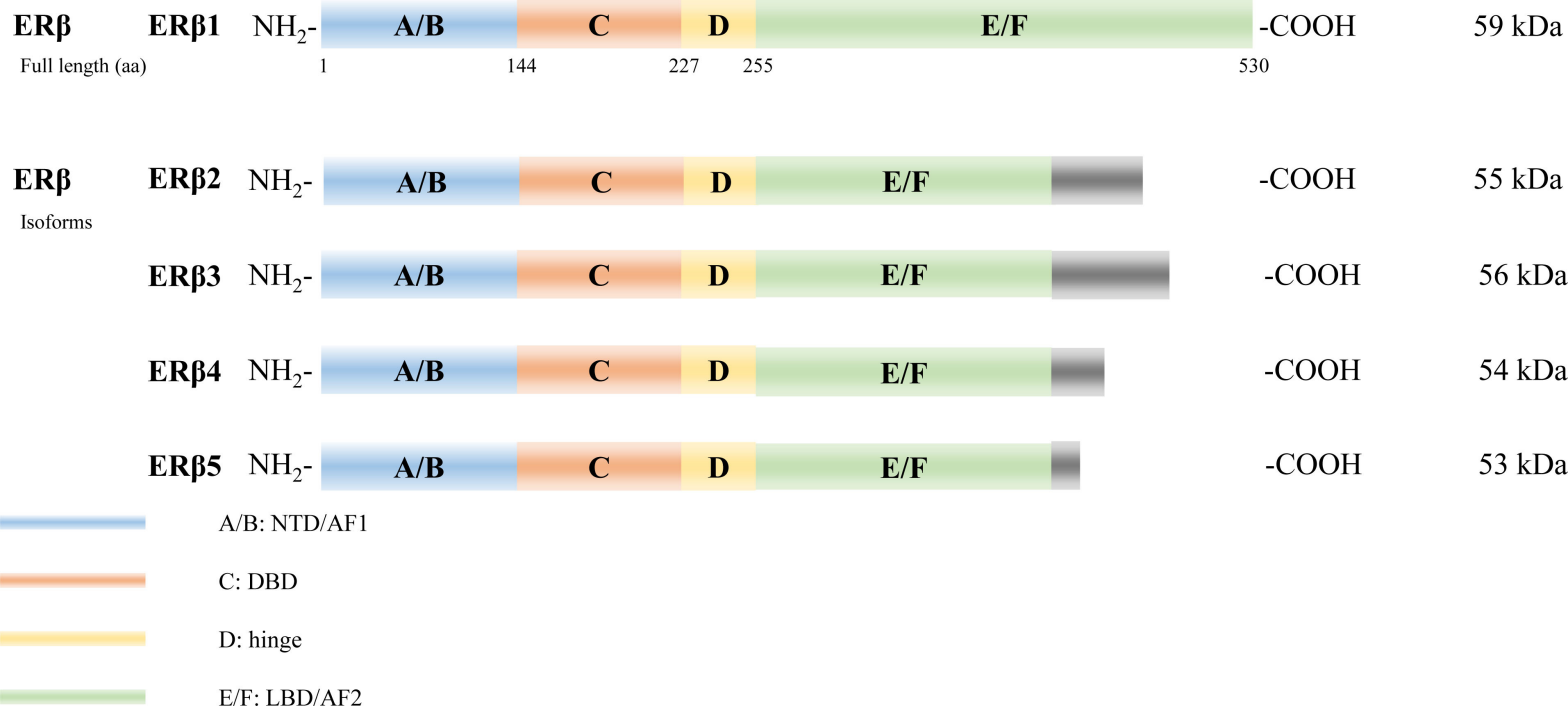
Estrogen receptor beta (ERβ) isoforms. Six structural and functional domains are highlighted: A, B domain (amino- or NH_2_-terminal domain [NTD], AF1), C domain (DNA binding domain [DBD]), D domain (hinge region connecting the C and E domain), E, F domain (carboxyl- or COOH-terminal, ligand-binding domain [LBD], AF2).

Several isoforms of ERα have been identified arising from alternative gene splicing, including ERα-46 (27) and ERα-36 (28) (**Figure 2**). ERα-46 lacks 1–173 amino acids, including AF1, and is a dominant-negative inhibitor of ERα activity in osteoblasts (29). ERα-36 lacks AF1 and AF2, and its unique 22 amino-acid sequence replaces the last 138 amino acids (30). Additionally, several ERβ splice isoforms have also been discovered (31, 32) (**Figure 3**). The full-length and truncated ERβ differ in their LBD. Particularly, ERβ1 (often referred to as ERβ) is a full-length construct that contains 530 amino acids. ERβ2-5 display unique LBD sequences. These differences result in truncation of the LBD and ablation of the AF2 function. Therefore, ERβ1 is the only isoform with ligand binding abilities, whereas the truncated ERβ is incapable of binding to estrogens and other investigated ligands (33).

**Figure 4:**
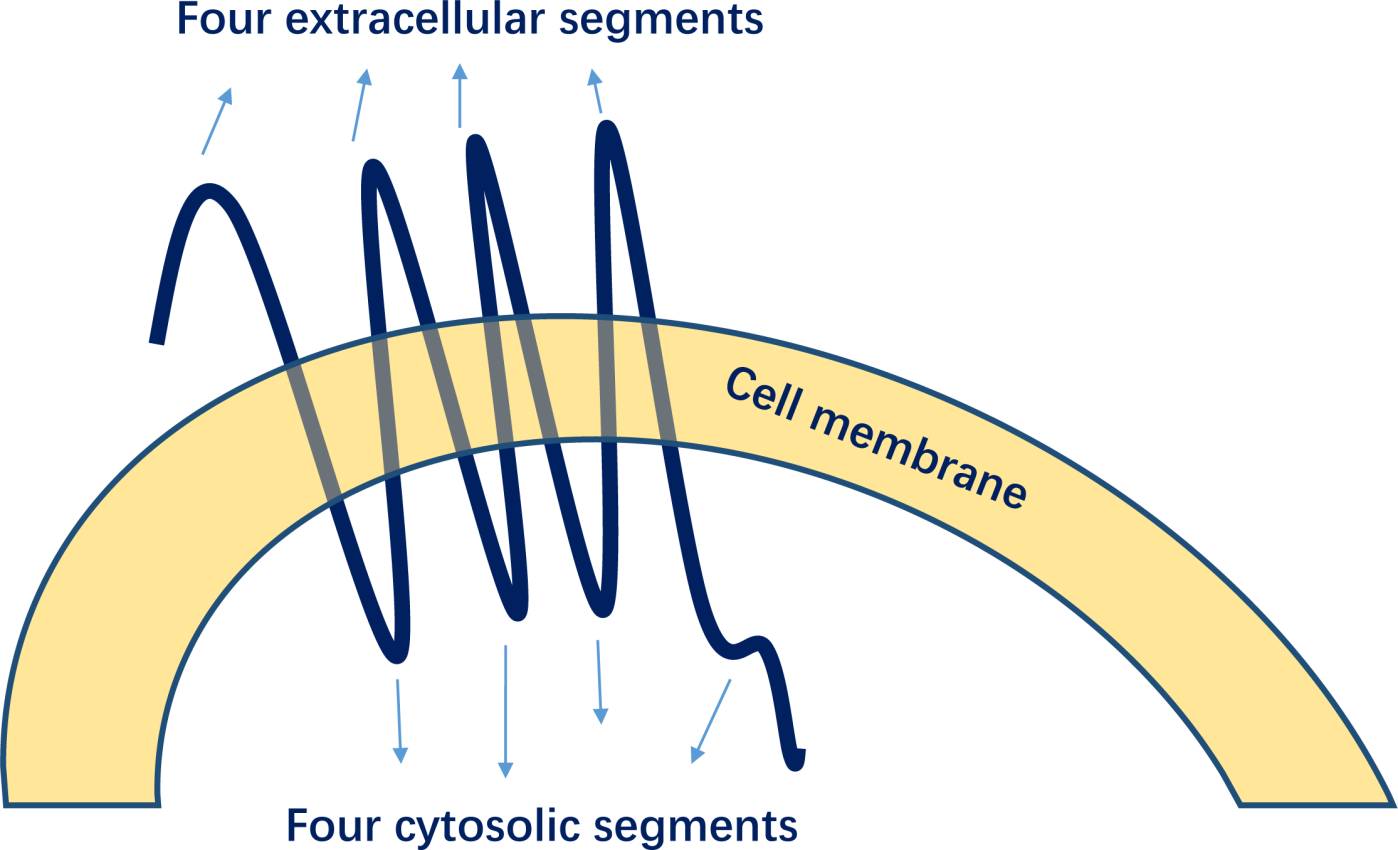
Structures of G-protein-coupled estrogen receptor 1 (GPER1).

GPER1 is encoded on chromosome 7 (7p22.3) and consists of 375 amino acids, with a molecular mass of 41 kDa (15). As a typical G protein-coupled receptor, GPER1 is distinct from ERα or ERβ and has a structure comprising seven transmembrane α-helices, four extracellular segments, and four cytosolic segments (**Figure 4**) (34). GPER1 demonstrates a weaker binding affinity to estrogens (35).

### ERs expression and signaling pathways

ERα, ERβ, and GPER1 are the three predominant ERs. ERα is primarily expressed in the reproductive tissues (e.g., uterus and ovary), bone, white adipose tissue, kidney, liver, and breast. In contrast, ERβ is expressed in male reproductive organs, central nervous system (CNS), cardiovascular system, lung, immune system, colon, and kidney (26). GPER1 is more widely distributed and expressed in the skeletal muscle, neurons, vascular endothelium, various immune cells, and target effector organs (36). Additionally, GPER1 is reportedly expressed in breast, ovarian, and lung cancer tissues (37).

**Figure 5:**
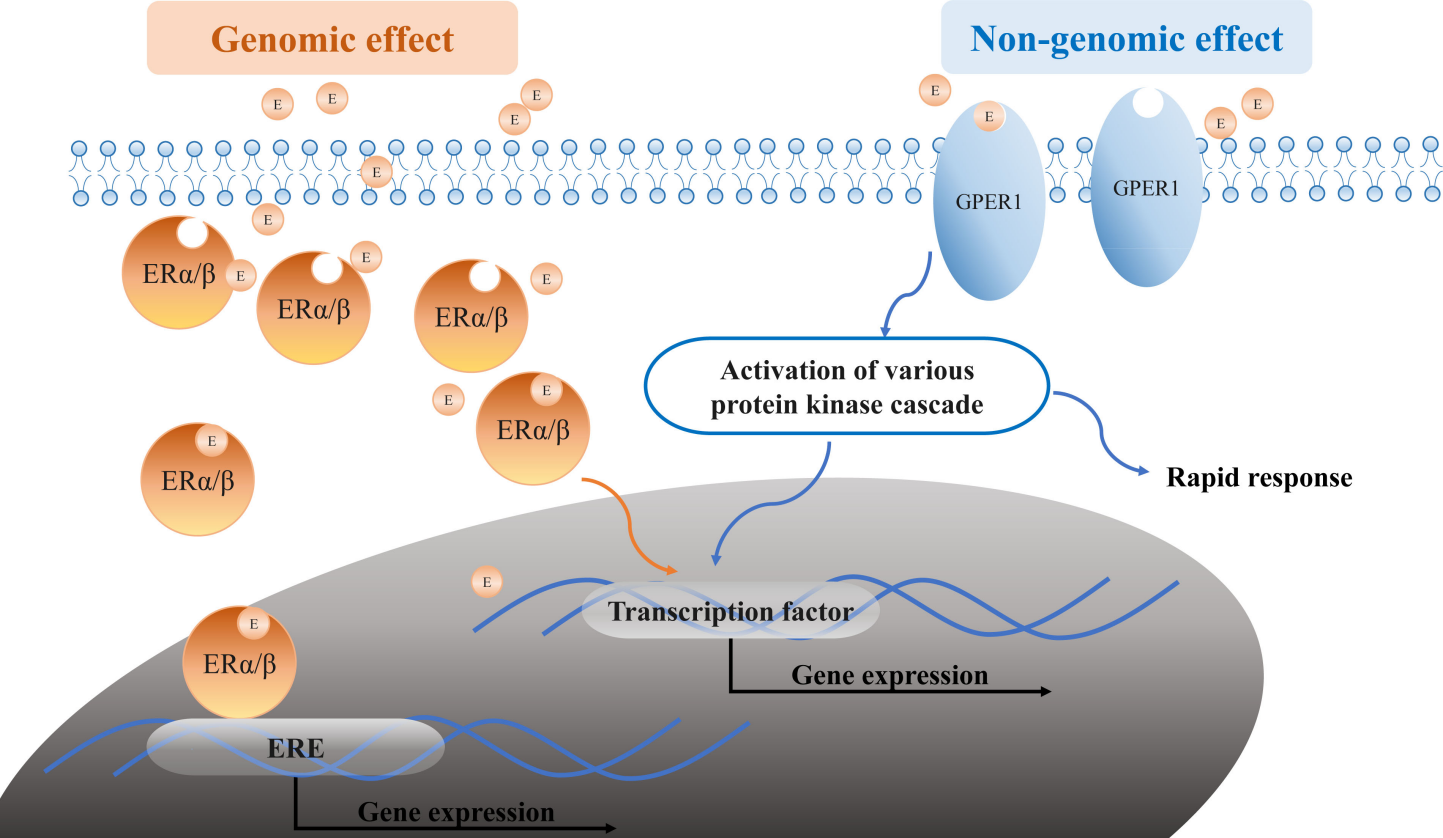
Estrogen signaling pathways. Estrogen or E2 (orange circle E in the graph) binds to the ERα/ERβ and GPER1, exerting its genomic and non-genomic effects. The genomic effect is shown in orange: the E2-receptor complex binds to EREs upon entry into the nucleus. The non-genomic effect is shown in blue: E2 binds to ERs in the membrane-like GPER1 and regulates the expression by modulating the ion channel opening or activation of related enzymes. E, estrogen or E2; ERα, estrogen receptor alpha; ERβ, estrogen receptor beta; GPER1, G protein-coupled estrogen receptor 1; ERE, estrogen response elements; PI3K, phosphatidylinositol 3-kinase; MAPK, mitogen-activated protein kinase.

Estrogens (for example, E2) bind to the traditional ERs (ERα and ERβ) and the novel receptor GPER1, exerting their genomic and non-genomic effects (**Figure 5**). In the genomic estrogen pathway, E2 binds to the intracellular ERα and ERβ and forms the E2-receptor dimer complex, subsequently entering the nucleus. In the nucleus, the complex binds to estrogen response elements (EREs) or activator protein-1 (Ap1) and specificity protein-1 (Sp1) on the E2-responsive gene promoters, acting as transcription factors that regulate gene transcription (1). Ultimately, estrogen-mediated gene products regulate autophagy, proliferation, apoptosis, survival, differentiation, and vasodilation under normal conditions. However, their function might be disrupted under pathological conditions. Owing to the intracellular location of the ERα and ERβ, the activation typically takes hours or more, leading to the slow “genomic effect.” E2 also mediates the non-genomic signaling pathway by binding to membrane-bound ERα, ERβ, and GPER1, which rapidly activates nuclear transcription factors by regulating the ion channel opening or the activation of related enzymes such as Ca^2+^ mobilization, phosphatidylinositol 3-kinase (PI3K), and mitogen-activated protein kinase (MAPK). This process does not rely on gene regulation and occurs instantaneously within a time span of seconds to minutes; thus, it is referred to as a rapid “non-genomic effect”.

Estrogens also regulate the immune response. ER signaling regulates hematopoietic progenitor populations during homeostasis and this likely regulates the number and type of immune cells (38). Present of E2 is necessary for hematopoietic stem cells (HSC) self-renewal. ERs are also widely expressed in many cell types involved in innate and adaptive immune responses (39). A published review suggests that ERα and ERβ mRNAs or proteins are expressed by hematopoietic progenitors and mature immune cells (40). In human tissue, the *ESR1* is highest expressed in the B cell, and intermediately expressed in CD4^+^ T cells, CD8^+^ T cells, NK cells, and plasmacytoid dendritic cell. Monocytes have the lowest levels of ESR1 RNA. Interestingly, *ESR1* expression level increase in monocyte-derived dendritic cells, suggesting that *ESR1* is induced during dendritic cell differentiation. *ESR2* have highest level in B cells and plasmacytoid dendritic cells, and lower levels in other cell types. GPER1 are also expressed in various immune cells like T cell, B cell, mononuclear, macrophage, and neutrophils (41). These evidences mean that estrogens can induce immune responses according to cellular expression of ER and the number of these cells. In addition, estrogens influence the adaptive immune response. Estrogens modulate the differentiation process and the function of neutrophils, macrophages, and natural killer cells (42).

## ERs in the manifestation of diseases

### ERs in breast cancer

Breast cancer is the most prevalent cancer affecting women worldwide. It is classified into three subtypes according to the expression status of ER, progesterone receptor (PR), and human epidermal growth factor receptor 2 (HER2) (1): luminal A/B (ER+PR+HER2-) (2), HER2-positive (ER-PR-HER2+), and (3) triple-negative (ER-PR-HER2-, abbreviated TNBC). Most breast cancers are luminal or ER-positive (approximately 60–80%) (43). ERs reportedly participate in numerous processes such as cell survival, proliferation, and tumor growth in breast cancer. Anti-estrogen therapy is considered the “gold standard” for treating luminal breast cancer (43). ERs affect the occurrence and development of breast cancer by binding to estrogens, thereby activating a unique signaling pathway.

ERα is overexpressed in breast cancer, which is approximately 10% in healthy tissues compared to 50–80% in breast cancer tissues (44). Additionally, ERα promotes tumor growth in breast cancer by interacting with estrogen. A study conducted in 1999 demonstrated the proliferative and differentiative roles of ERα in mammary gland development (45). ERα functions as a transcription factor for genes associated with tumor cell proliferation and growth, such as insulin-like growth factor-1 receptor (IGF1R), cyclin D1, anti-apoptotic BCL-2 protein, and vascular endothelial growth factor (VEGF). The phosphorylation of ERα serine 118 and serine 167 in breast cancer cells induces unique gene expression profiles, ultimately impacting tumor growth and morphology as well as the hormonal therapy responsiveness in patients with breast cancer (46). Anti-hormonal therapy with drugs such as tamoxifen has been widely used to treat patients with breast cancer. It exerts its effects by suppressing ERα-mediated transcriptional regulation and is used as first-line therapy. However, a significant proportion of the affected individuals eventually relapse and develop resistance to this antagonist (47). Tamoxifen can cause severe side effects in some tissues, such as those in the skeletal and cardiovascular systems, owing to its anti-estrogenic properties. Raloxifene has fewer side effects than tamoxifen. Other drugs targeting ERα, such as toremifene, fulvestrant, anastrozole, letrozole, and exemestane, have also been clinically approved for breast cancer therapy (26). Moreover, ERα splice variants are also implicated in breast cancer. ERα-46, is expressed in 70% of breast cancer tissues and occasionally shows a higher expression level than the full-length ERα-66. ERα-46 acts as a competitive inhibitor of ERα when co-expressed with ERα-66 (27). ERα-36, which lacks both AF1 and AF2, is considered a membrane-based ER that regulates membrane-initiated non-genomic signaling (28, 30). Tamoxifen acts as an agonist of ERα-36 in breast cancer and contributes to hormone therapy resistance and metastasis (48).

However, the role of ERβ in breast cancer remains controversial. The ERβ expression level decreases during breast cancer by approximately 80% in healthy tissues (44). ERβ knockdown does not affect mammary gland development. However, ERβ is occasionally associated with increased proliferation in breast cancer tissues (49). *In vitro* studies revealed that the re-expression of ERβ in breast cancer cells repressed cell proliferation, promoted cell apoptosis, and sensitized the tumor cells to chemotherapy (50). In addition, clinical studies have demonstrated that the absence of ERβ results in a poor prognosis (51) and resistance to hormonal therapy (52). In breast cancer, ERβ inhibited cell proliferation by suppressing the activation of MAPK and PI3K signaling pathways (53). However, a few reports support the notion that ERβ is a poor prognostic factor in breast cancer, and its expression is related to enhanced cell proliferation (54, 55). Some researchers have suggested that ERβ expression is not associated with clinical outcomes in women who have undergone menopause (56). Therefore, further experiments are required to validate our current understanding of the link between ERβ and breast cancer. Moreover, ERβ2 and ERβ5 might sensitize the breast cancer cells to therapy, thus leading to a good prognosis (57). ERβ2 is also sensitive to hormonal therapies. Although ERβ2 and ERβ5 cannot form functional homodimers, they can heterodimerize with ERα, thereby inhibiting the ERα function (26). We speculate that this might be the putative basis for their protective mechanism. Notably, ERα and ERβ form functional heterodimers when co-expressed in tissues (33). In this scenario, ERβ might inhibit the ERα-mediated gene expression.

GPER1 is thought to play a favorable role against breast cancer. In patients with breast cancer, GPER1 downregulation in the tumor tissue is associated with poor survival (58). GPER1 is detected in 60% breast cancer tissue. Among them, GPER1 expression has been confirmed in most TNBC, and the combined expression of GPER1 and ER accounts for about 40% of all cases (59). In contrast, GPER1 expression has been associated with reduced response or resistance to tamoxifen therapy in patients with breast cancer, mediated by regulating HMGB1 (high mobility group box 1) (60, 61). Recently, a study identified GPER1 as a crucial therapeutic target for triple-negative breast cancer, as it elicits an NF-κB/IL-6 signaling inhibition-mediated suppression of migration and angiogenesis (62).

**Figure 6:**
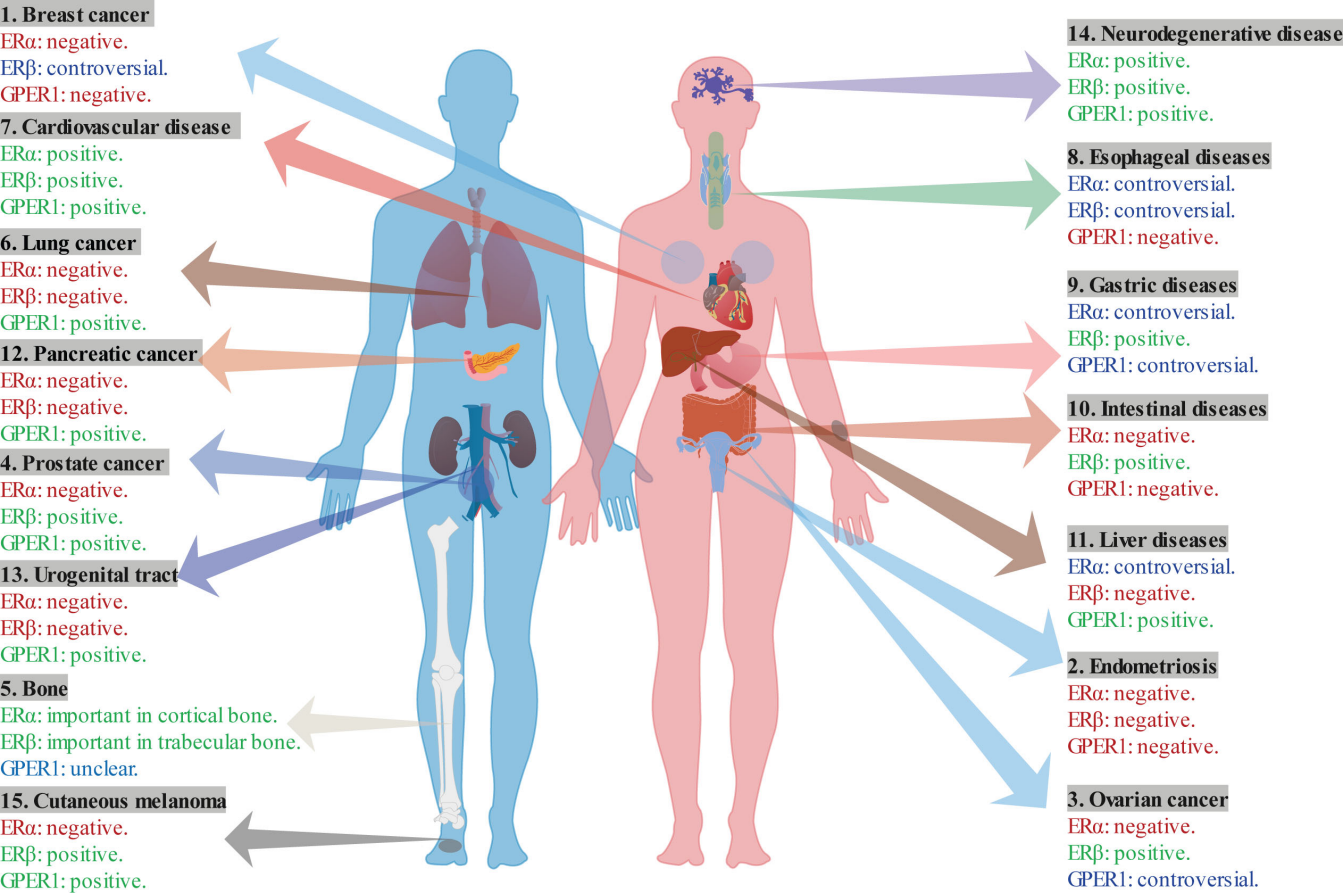
The multifaceted role of ERs in various diseases.

In conclusion, ERα and GPER1 show promising roles in breast cancer development, whereas the role of ERβ remains controversial (**Table 1**; **Figure 6**). Specially, ERβ acts as a protective role through inhibiting the function of ERα. Moreover, ERα-46 is a competitive inhibitor of the full-length ERα. ERα-36 is associated with resistance to hormonal therapy. ERβ2 and ERβ5 play protective roles in breast cancer.

**Table 1:** The role of ERs in various diseases.

Number	Disease	ERα	ERβ	GPER1
1	Breast Cancer	ERα: promotes tumor ◼ (45).	ERβ: inhibits tumor ● (50, 53), promote tumor ● (54, 55); ERβ2 and ERβ: acting as protective role through inhibiting the ERα ● (26, 57).	GPER1: inhibits tumor migration and angiogenesis ● (62), promote tamoxifen resistance ● (60, 61).
2	Endometriosis	ERα: promoting endometriotic-like lesions ◼ (63).	ERβ: promotes tumor ◼ (64).	GPER1: promote tumor ● (65).
3	Ovarian Cancer	ERα: promoting ovarian cancer ● (66).	ERβ: inhibits tumor ● (66); inhibits the ERα ◼ (67). ERβ2 and ERβ5: promotes tumor ● (68, 69).	GPER1: inhibits tumor ● (70), promote tumor ● (71–73); GPER1 relies on ERα expression ● (74).
4	Prostate Cancer	ERα: promote cell proliferation ♦◼ (75–77).	ERβ: inhibits tumor ◼ (77), represses ERα ◼ (77, 78); ERβ5: promote tumor ● (79).	GPER1: inhibit tumor ◼ (77, 80, 81).
5	Bone	ERα is expressed in osteoblast, osteocytes, and osteoclast ● (82). ERα in cortical bone is higher than in trabecular bone ● (82); ERα: important in cortical bone ◼ (83).	ERβ is expressed in osteoblast, osteocytes, and osteoclast ● (82). ERβ in trabecular bone is higher than in trabecular bone ● (82); ERβ: important in trabecular bone ◼ (84, 85).	GPER1 is expressed in osteoblasts, osteocytes, and osteoclasts ● (86); Loss of GPER1: decreases the bone growth in female mice ◼ (87), increases the bone mass in male mice ◼ (88).
6	Lung Cancer	ERα is mainly expressed in basal and smooth muscle cell ● (89). ERα: promotes tumor ● (90).	ERβ is mainly expressed in columnar epithelium and intermediate, basal and smooth muscle cells ● (89). ERβ: promote tumor ◼ (91).	GPER1: inhibits tumor ◼ (92).
7	Cardiovascular Disease	ERα is highly expressed in PASMCs and VSMCs ◼● (93, 94); ERα improves cardiac recovery ●◼○♦ (94–101).	ERβ is expressed in endothelial cells and VSMCs of arteries ● (102); ERβ: improves cardiac recovery ◼♦ (95, 103–106), decreases fibrosis, inflammation, vasoconstriction, and right ventricle hypertrophy ◼♦ (107–111).	GPER1 is widely distributed in the cardiovascular system ♦ (112); GPER1: improves cardiac recovery ◼♦ (113–117), decreases cell proliferation of fibroblasts and VSMC ♦ (118).
8	Esophageal Diseases	ERα inhibits the tumor in ESCC ● (119, 120); ERα promotes tumor ● (121).	ERβ inhibits the EC ● (122, 123); ERβ promotes EC ● (124).	GPER1: promotes the tumor ● (125).
9	Gastric Diseases	ERα: inhibits tumor ● (126); promotes tumor ● (127); ERα-36: promotes tumor ● (128).	ERβ: inhibits tumor ● (129, 130); ERβ5: promotes tumor ● (131).	GPER1: inhibits tumor ● (132), promotes tumor ● (133).
10	Intestinal Diseases	ERα: promote tumor ● (134).	ERβ: inhibits tumor ●◼ (135–137).	GPER1: induce pain severity in IBS ●◼ (138, 139).
11	Liver Disease	ERα: inhibits the liver cancer ● (140–142). Promote liver cancer ● (143).	ERβ: inhibits tumor ◼● (144, 145).	GPER1: inhibits tumor●◼ (146, 147).
12	Pancreatic Disease	ERα: promotes tumor ◼ (148).	ERβ: promotes tumor ◼ (149).	GPER1: inhibits tumor ♦● (150, 151).
13	Urogenital Tract Disease	ERα: promotes tumor ● (152).	ERβ: promotes tumor ◼ (153).	GPER1: inhibits tumor ● (154).
14	Neurodegenerative Disease	ERα is expressed in cortical and hippocampal neural stem/progenitor cells. ERα levels in female are higher than that in male ● (155). ERα is associated with the regulation of reproductive functions, including the hypothalamus and preoptic region ◼ (156).	ERβ is widely distributed and expressed in the hippocampus and cerebral cortex, lateral septa, and medial and basolateral amygdala. ERβ expression levels are higher than that of ERα in hippocampi ● (155); ERβ promotes the survival and differentiation of brain neurons ◼ (157, 158).	GPER1 is expressed in cortical and hippocampal neural stem/progenitor cells ● (155). GPER1 increase dendritic spine density in the hippocampus and protect the nerve ◼♦● (159–161).
15	Cutaneous Melanoma	ERα: promote the melanoma ◼ (162).	ERβ: inhibits the melanoma ● (163).	GPER1: inhibits the melanoma ●◼ (164, 165).

●, study in human or human cell line; ◼, study in mouse; ♦, study in rat; ○, study in rabbit; PASMCs, pulmonary artery smooth muscle cells; VSMCs, vascular smooth muscle cells; ESCC, esophageal squamous cell carcinoma; EC, esophageal cancer; IBS, irritable bowel syndrome.

### ERs in endometriosis and ovarian cancer

Endometriosis, characterized by lesions of endometrial-like tissue outside the uterus, is a chronic inflammatory disease with a phenotype similar to that of ovarian cancer. Endometriosis and ovarian cancer are hormone-dependent gynecological diseases with severe consequences on fertility and daily routine in women (166). Ovaries are the primary source of estrogen and progesterone in women. ERs affect the occurrence and progression of endometriosis (167) and ovarian cancer. In this section, we discuss the role of ERs in endometriosis and ovarian cancer.

The expression of ERα is higher than ERβ in the normal endometrium (168). Compared to normal endometrium, ectopic tissues show menstrual cycle-dependent increased ERα expression (169, 170). A study conducted on ERα-knockout mice suggested that ERα participates in the formation of endometriotic-like lesions (63). ERα likely promotes ovarian cancer development. ERα and ERβ are expressed in most patients with ovarian cancer (80%) (66). ERα is a contributing factor in developing ovarian cancer by promoting cell proliferation and migration (66). Multiple clinical studies have shown that ERα responds to hormonal therapies such as tamoxifen and aromatase inhibitors (66), which inhibit the conversion of androgen to estrogen, thereby reducing the circulating estrogen levels (66). Extensive studies have been conducted on ERα, which is reportedly mediated by long non-coding (lnc) RNAs that subsequently play a role in promoting ovarian cancer development (171). The lncRNA MIR2052HG regulates the ERα expression and confers resistance to aromatase inhibitors through lemur tyrosine kinase-3 (LMTK3) by recruiting early growth response protein 1 (EGR1) (172). The ERα expression and its role in promoting ovarian cancer progression suggest that hormonal therapy could be a viable treatment option. However, anti-estrogen therapy has a modest response rate among patients with ovarian cancer and is, therefore, not a commonly prescribed therapy (26).

ERβ expression is elevated in ectopic tissue than in the normal endometrium (169). In a mouse model of endometriosis, elevated levels of ERβ were detected in both the nucleus and cytoplasm (173). The specific mechanisms underlying the increased ERβ levels remain unclear. Hypermethylation of the ERβ promoter region could be associated with increased protein levels in the endometrial tissues (174). Methylation anomaly induces aberrant expression of GATA family members, which regulate the uterine physiology, and subsequently promotes abnormal expression of several genes, such as steroidogenic factor 1 (*NR5A1*) and aromatase (*CYP19A1*) (167, 175). Another mouse model-based study suggested that ERβ inhibits tumor necrosis factor α (TNF-α)-induced apoptosis by interacting with the cytoplasmic apoptotic machinery. The authors further demonstrated that the combined action of ERβ and the cytoplasmic inflammasome components result in the subsequent increase in interleukin (IL)-1β levels and promote cell survival, cellular proliferation, invasion, and adhesive activities of endometrial cells (64). These results indicate that ERβ overexpression facilitates the progression of endometriosis. In contrast, ERβ is generally regarded as a tumor suppressor in ovarian cancer and is associated with inhibiting cell growth and invasion (66). Decreased ERβ levels or ERβ/ERα ratio during ovarian carcinogenesis also suggests that the loss of ERβ might play a role in cancer progression. Treatment with the ERβ agonist diarylpropionitrile (DPN) or re-expression of ERβ significantly suppressed ovarian cell growth (26). Furthermore, ERβ direct affects ERα by strongly inhibiting its expression and activity, thereby exerting an anti-proliferative activity (67). In contrast to the inhibitory effect of full-length ERβ, both ERβ2 and ERβ5 isoforms are associated with pro-migratory and invasive activities in ovarian cancer (68). Cytoplasmic ERβ2 expression is associated with poor overall survival in patients with high-grade serous ovarian cancer (HGSOC) (69). High nuclear ERβ5 expression levels have also been observed in advanced ovarian cancer, especially in serous and clear cell carcinomas, and are associated with poor survival (68).

Additionally, estrogens can exert their effects through non-genomic signaling *via* mERs. GPER1 expression is reportedly higher in ovarian endometriosis than in the normal endometrium, and this expression is associated with estrogen levels and the extent of inflammation (176). This overexpression is also observed in endometriomas (177). Treatment with the GPER1 agonist G1 stimulated endometriotic cell proliferation as well as rapid Akt phosphorylation. In contrast, treatment with antagonist G15 reversed this proliferation and caused Akt dephosphorylation (65). Collectively, these results suggest a stimulatory role for activated GPER1 in the growth of ectopic endometrial tissues. However, GPER1 plays a complex role in ovarian cancer, demonstrating stimulatory and suppressive functions in cancer cells (66). Ignatov et al. considered GPER1 a tumor suppressor in ovarian cancer owing to its lower expression in ovarian cancer tissues than in benign tissues. The higher GPER1 expression in the early stages of ovarian cancer further emphasized its anti-tumor role (70). In contrast, Smith et al. and Zhu et al. demonstrated that GPER1 was associated with poor survival in patients with ovarian cancer (71, 72). In ERα-negative ovarian cancer cells, the G1 agonist stimulated the tumor cell proliferation and increased the number of cells in the S phase (72). Yan et al. highlighted the GPER1 ligand-independent stimulation mechanism in ovarian cancer cell proliferation, migration, and invasion (73). Albanito et al. proposed an interactive effect of ERα expression-dependent GPER1/epidermal growth factor receptor (EGFR) signaling on the growth response (74).

In summary, ERα, ERβ, and GPER1 promote endometriosis (**Table 1**; **Figure 6**). Nevertheless, ERα tends to be a promoting factor, whereas ERβ protects against ovarian cancer progression. The role of GPER1 tends to be more complex, displaying both promoting and protective effects in these disorders. Furthermore, ERβ2 and ERβ5 isoforms show a protective function in ovarian cancer. Therefore, further investigations on the role of ERs in gynecological disorders should be conducted to improve our understanding of the underlying molecular mechanisms.

### ERs in prostate cancer

Prostate cancer, an epithelial malignancy of the prostate gland, is responsible for the highest number of cancer-related deaths in men. Its incidence increases with age. Several studies have demonstrated strong associations between ERs and prostate cancer, including their occurrence, development, and prognosis. However, the role of ERs in castration-resistant prostate cancer (CRPC) progression remains unclear. Serum estrogen levels are associated with the risk of prostate cancer development. This indicates that estrogen and ERs, which mediate the action of estrogens, may be risk factors (178, 179).

ERα is expressed in stromal tissues of the human prostate (180). The stromal cell receptor ERα stimulates the growth of prostatic epithelium *via* growth factors such as basic fibroblast growth factor (bFGF), epidermal growth factor (EGF), and IGF-1 (181). Data from animal models have revealed the indirect tumor-promoting role of ERα in the prostate epithelium. bFGF and EGF in the ventral prostate of adult rats and IGF-1 in the monkey prostate stimulated the prostatic epithelial cell proliferation (75, 76). Animal studies have shown that E2 and its association with testosterone induce prostate lesions and cancer (77). Combinatorial treatment with testosterone and E2 prevented the development of high-grade prostatic intraepithelial neoplasia (HGPIN) or prostate cancer following ERα knockdown (182). These observations indicate that ERα is crucial in the development of prostate cancer. ERα expression is upregulated in high-grade prostatic intraepithelial neoplasia (11%, 43%, 61%, and 94% in HGPIN, Gleason grade 4, grade 5, and recurrent adenocarcinoma, respectively, after therapy) (183).

In benign prostates, ERβ is primarily located in the cytoplasm and nucleus of basal epithelial cells as well as in the perinuclear region of luminal epithelial cells. In contrast to ERα, ERβ plays a tumor-suppressive role in cancer development and progression. ERβ is primarily expressed in epithelial cells (180), whereas other studies have demonstrated that ERβ is undetectable in stromal and epithelial cells (5). E2 exerts ERβ-mediated direct effects on the prostate epithelium. ERβ reportedly inhibits androgen receptor (AR) signaling, inflammation, and cell proliferation by downregulating AR signaling, inducible nitric oxide synthase (NOS), antioxidant glutathione peroxidase 3 gene, and interleukin (IL)-6. In contrast, it upregulates the tumor suppressor phosphatase and tensin homolog (PTEN) in the mouse ventral prostate (77). ERβ activation can suppress the effects of ERα and induce apoptosis in prostate cancer cells (77, 78, 184). The inhibitory role of ERβ is further supported by higher ERβ levels in primary prostate cancer, whereas ERβ is suppressed in high-grade prostate cancer (185). Markedly reduced ERβ levels can be observed in grade 4/5 carcinomas compared to that in grade 3 carcinomas (186). Five splice variants of ERα and ERβ (ERα-36, ERβ2, ERβ3, ERβ4, and ERβ5) were detected in men with and without prostate cancer (187). However, the role of ERα-36 remains unclear. In contrast to ERβ2, ERβ5 is almost entirely located in basal epithelial cells, underscoring its carcinogenic role (79).

The significance of GPER1 expression and signaling in prostate cancer biology remains unclear. GPER1 might exhibit a non-genomic estrogenic response together with ERα or ERβ (181). Contrary to its promoting function in breast cancer and endometriosis, GPER1 activation inhibits the growth of normal and malignant bladder urothelial cells (154). The suppressed GPER1 function has been demonstrated to aid the growth of prostate cancer cells. GPER1 also plays a role in sustainably activating erk1/2, c-Jun/c-fos-dependent upregulation of p21, and induction of G2 cell-cycle arrest (80). Increasing evidence suggests a protective function for GPER1 in prostate cancer. GPER1 knockout weakens these protective effects (81). A recent study also reported that treatment with G1 agonist induces massive tumor necrosis in castrated mice with CRPC (77).

In conclusion, the three ERs have distinct effects on prostate cancer, wherein ERβ and GPER1 exert tumor growth-suppressive effects (**Table 1**; **Figure 6**), and ERα, ERβ2, and ERβ5 exert tumor growth-promoting effects.

### ERs in bone

Estrogens are vital for maintaining bone mineral density in humans. Osteoblasts and osteoclasts are involved in synthesizing the bone matrix and its degradation, respectively. E2 exhibits a protective role in the bone remodeling process by increasing the bone mass by inhibiting pro-osteoclastic cytokines in T cells and promoting the anti-apoptotic activity in osteoblasts (188, 189). The estrogen levels decline in women undergoing menopause, leading to a decreased bone density and increased risk of fractures (190). Hormone replacement therapy prevents this process through the mediation of ERs.

ERα and ERβ are highly expressed in osteoblasts, osteocytes, and osteoclasts (82). They are also expressed in immune cells, which are essential for bone cell regulation (40, 191, 192). The level of ERα is higher in cortical bone than trabecular bone, whereas the converse is true for ERβ (82), suggesting that ERα and ERβ may have opposing functions in these tissues. A mouse model study shows that ERα-knockout decreased cortical bone mineral density and increased that of the trabecular bone (83). In ERβ-knockout mice, cortical bone mineral density increases at 11 weeks of age, and both cortical and trabecular bone mineral density increases at 12 months of age (84, 85). The higher trabecular bone density in ERβ-knockout mice than in the ERα-knockout mice (85) suggests that ERβ plays a vital role in trabecular bone formation. The opposing effects of ERα and ERβ on the femoral bone length have also been demonstrated, with the ERβ-knockout mice developing longer femur bones than the ERα-knockout mice (193). Bone cells from ERβ-knockout mice cultured under mechanical strain showed an increase in osteoblast-like cells, whereas this increase was not observed in cells cultured from ERα-knockout mice (194). This underscores the role of ERα in augmenting the response of bone cells to mechanical strain, whereas ERβ exerts a suppressive function in this process.

Relatively few studies have been conducted on GPER1. Although GPER1 expression has been reported in osteoblasts, osteocytes, and osteoclasts (86), their role in bone growth remains controversial. Loss of GPER1 might lead to bone growth reduction in female mice (87). In male mice, GPER1 deficiency increases bone mass, mineralization, and growth plate proliferative activity (88). GPER1 activation protects the bones of ovariectomized rats from developing osteoporosis and has no adverse effects on the uterus (195). However, G1 agonist-mediated stimulation does not influence bone growth in mice (196).

In conclusion, ERs play crucial yet opposing roles in mediating bone homeostasis. ERα is important in the cortical bone, whereas ERβ is vital in the trabecular bone (**Table 1**; **Figure 6**). ERα is also involved in mechanical strain responses. GPER1 might be associated with bone growth. However, additional studies should be conducted to determine their precise role in these processes.

### ERs in lung cancer

Lung cancer is the leading cause of cancer-related death worldwide. Estrogen is presumed to play an important role in lung cancer development (197). Approximately 85–90% of lung cancer cases are attributed to smoking (198). Women are at a higher risk than men to develop lung cancer regardless of their smoking status. The higher susceptibility of women to the adverse effects of tobacco could be owed to estrogen-mediated responses (197). Older women with lung adenocarcinoma have a significant survival advantage compared to their male counterparts, possibly owing to the reduced effectiveness of estrogens in promoting cancer (199). Estrogens, which are mediated by ERs, adversely affect the prognosis of patients with lung adenocarcinoma.

ERα and ERβ are both expressed in the health and normal lung tissue (200). The expression level of ERα in lung cancer remains unclear, while ERα mainly resides in the cytoplasm of lung cancer cells and is associated with a poor prognosis (90, 201). It reportedly promotes cell invasion in lung cancer, mediated by increased cross-talk with the infiltrating macrophages (90).

ERβ is highly expressed in pneumocytes and bronchial epithelial cells in healthy lung tissue and is required to maintain its extracellular matrix (202, 203). ERβ-deficient mice have fewer alveoli and a lower amount of surfactant (204). ERβ appears to be dominant in lung cancer, especially in adenocarcinoma (205). It is also present in adenocarcinoma tissues, and the absence of ERβ tends to result in poor overall survival (201). Most reports have suggested that nuclear ERβ is associated with a better prognosis, whereas its cytoplasmic form is associated with a worse prognosis (206, 207). A recent study showed that ERβ promotes lung cancer invasion by increasing C-X-C chemokine receptor type 4 (CXCR4) expression (91). Among the five identified splice variants, ERβ1 is the only full-length receptor capable of binding ligands and forming homodimers in humans. Although the other isoforms are inactive, they form heterodimers with ERβ1 to regulate their transcriptional activity (208). Overexpression of ERβ5 and EGFR is reportedly associated with poor prognosis and reduced overall survival in patients with non-small cell lung cancer (NSCLC) (209).

Increased GPER1 expression, especially in the cytoplasm, has been observed in lung cancer cells (92, 210). GPER1 is likely involved in the proliferation, migration, and invasion of cancer cells (211). GPER1 agonist G-1 play an antiproliferative and proapoptotic effects in lung cancer (212).The *in vitro* observations from the E2 and G1 models suggested that an increase in tumor nodules, grade, and the index could be reversed by co-administration of GPER1 inhibitor G15 (92).

In conclusion, ERα is mainly expressed in basal and smooth muscle cell, and exhibits as a promoting factor in lung cancer progression (**Table 1**; **Figure 6**). ERβ is mainly expressed in columnar epithelium and intermediate, basal and smooth muscle cells, and shows a promoting role in lung cancer. In addition, exist of ERβ in different cell areas shows different prognosis. As for GPER1, the present study shows that it exhibits an inhibiting role in tumor growth and migration. ERs in cardiovascular diseases

Cardiovascular diseases (CVD) are a collective group of disorders caused by abnormalities in the heart and blood vessels. They are the principal cause of death in both men and women. Epidemiologic studies have shown that prior to menopause, women are at a lower risk of developing CVD compared to men. Women also have decreased CVD-associated morbidity compared to age-matched men, and present with CVD 10 years later than men (213). Heart failure-related morbidity is reportedly higher in men at any age (214). Women who are pre-menopausal are at reduced risk of ischemia/reperfusion (I/R) injury compared to men (215). Women who have undergone menopause demonstrated worse survival than women who were premenopausal and age-matched men. Estrogen levels in women who have undergone menopause are negatively associated with the risk of CVD development (213). A recent study confirmed the protective effects of exogenous E2 on preexisting advanced heart failure in mice (104). Collectively, sex hormones, especially estrogens, tend to be protective factors in mediating homeostasis in patients with CVD, following the sex-related bias in CVD development. Estrogens mediate their functions by binding to ERs, which are also present in the cardiac tissues. In the following section, we explore the crucial roles of ERs in cardiovascular diseases.

#### Cardiac disease

Myocardial I/R injury and heart failure are two common heart-related diseases (213). I/R injury relates to the tissue damage caused by myocardial reperfusion following a period of ischemia, which is characterized by inflammation, oxidative stress, intracellular and mitochondrial calcium overload, apoptosis, or necrosis. Heart failure results from dysfunctional systolic and/or diastolic processes, manifesting as venous system blood obstruction and arterial system blood perfusion inadequacy. ERα, ERβ, and GPER1 protect against I/R injury and heart failure (213, 216).

Experiments on animal models have demonstrated the cardioprotective role of ERα in regulating I/R injury (217). ERα-knockout female rabbits exhibited significantly higher functional recovery than wild-type females, with similar effects compared to wild-type males. A study on rabbits also highlighted the cardioprotective role of ERα but not ERβ (96). Treatment with PPT (4, 4’, 4’’-[4-propyl-(1H)-pyrazole-1, 3, 5-triyl] tris-phenol, an ERα-selective agonist) in an I/R injury rabbit model decreased the infarct size. ERα-knockout male mice demonstrated reduced mitochondrial respiratory function, decreased nitrite production, and increased Ca^2+^ accumulation compared to control mice (97). Additionally, 16α-lactone-estradiol (16α-LE2), an ERα-selective agonist, reduced the progression of myocardial hypertrophy and systolic dysfunction during heart failure (98). Increased ERα expression also improved cardiac intercalated disk stability during heart failure in humans (99).

ERβ-knockout in mice induces severe heart failure and death, underscoring the protective role of ERβ (103). Treatment with ERβ agonist DPN improved cardiac function in mice by stimulating cardiac angiogenesis, suppressing fibrosis, and restoring hemodynamic parameters (104). ERβ is also essential for improving cardiac recovery after I/R injury by inhibiting apoptosis and preserving mitochondrial integrity (95). ERβ protects against cardiac hypertrophy, the primary risk factor for heart failure. ERβ knockout induces inflammatory pathways and modulates mitochondrial bioenergetics- and oxidative stress-related pathways (105). ERβ also suppresses cell death by inhibiting p53, thereby attenuating reactive oxygen species (ROS) in mitochondria-centered apoptotic processes (106).

GPER1 can protect the heart against I/R injury by inhibiting mitochondrial permeability transition pore (MPTP) opening (113). Another study reported enhanced protective effects of GPER1 in mitochondria, indicating that it can maintain mitochondrial structural integrity and function, thereby reducing mitophagy in I/R injury (114). Oxidative stress affects cardiac remodeling after ovarian estrogen loss. GPER1 deficiency induces cardiac remodeling through oxidative stress (115). Numerous reports have also revealed that GPER1 can decrease fibrosis and improve myocardial relaxation and diastolic function (116). The GPER1 agonist G1 reduced the risk of isoproterenol-induced heart failure in female mice (117). Treatment with G1 improved cardiac function and myocyte contractility, and reduced fibrosis. The cardioprotective ability of GPER1 is mediated by the expression of adrenergic receptors in ventricular myocytes.

In conclusion, ERα, ERβ, and GPER1 contribute to myocardial protection following I/R injury (**Table 1**; **Figure 6**). ERα exhibits functions of reducing infarct size, decreasing ROS production, and attenuating myocyte apoptosis. ERβ can attenuate apoptosis and decrease ROS production. Activation of GPER1 maintains mitochondrial function, decreases fibrosis, and improve myocardial relaxation and diastolic function.

#### Vascular disease

Hypertension (HTN), pulmonary hypertension (PH), and atherosclerosis are typical vascular diseases (213), wherein ERα, ERβ, and GPER1 are known to mediate protective functions. ERα activation reduces endothelial dysfunction by contributing to endothelial progenitor cell activation and VEGF upregulation (218). ERα is highly expressed in pulmonary artery smooth muscle cells (PASMCs), wherein it increases the proliferation of PASMCs *via* MAPK and Akt signaling and enhances vascular remodeling (93). ERα-knockout mice displayed lower recovery of cardiac function and greater difficulty in breathing (97). ERα expression reduced vasoconstriction and induced right ventricular (RV) hypertrophy in male mice (100). Loss of ERα increased the occurrence of atherosclerotic lesions through elevated serum cholesterol levels and increased high-density lipoprotein particle size (101). ERα tended to decrease vascular smooth muscle cell (VSMC) proliferation through reduced ROS-mediated extracellular signal-regulated kinase (ERK) phosphorylation (94). ERα also demonstrated anti-atherosclerotic properties such as increased antioxidation (219) and decreased lipid accumulation (220).

ERα and ERβ are both expressed in endothelial cells and VSMCs of arteries (102, 221). ERβ activation reduced endothelial dysfunction by activating endothelial progenitor cells (218), decreasing VSMC differentiation (222), and upregulating VEGF levels (218). ERα and ERβ expressions suppress apoptosis and eliminate the generation of intracellular ROS by decreasing the protein levels of ROS-generating enzymes, as well as preventing NF-κB activation in human aortic endothelial cells stimulated by H_2_O_2_ (107). In addition, estrogen rescued pre-existing severe PH by restoring the lung and RV structure in rats. The rescue ability was associated with the stimulation of neoangiogenesis, suppression of inflammation, fibrosis, and RV hypertrophy. These estrogen rescue effects are mediated by ERβ (108). ERβ-mediated increases in inducible NOS expression decrease vasoconstriction (109). ERβ decreased fibrosis, inflammation, vasoconstriction, RV remodeling, and RV hypertrophy (110, 111). ERβ has also been shown to decrease calcification (222).

GPER1 is widely distributed in the cardiovascular system and exerts protective effects on the vasculature (112). GPER1 decreases blood pressure, promotes vasodilation, and reduces the proliferation and migration of VSMCs (223). In addition, activation of GPER1 may limit fibroblast proliferation by inhibiting cyclin, cyclin B1, and CDK1 (118). E2-induced decrease in VSMC proliferation rate after injury is mediated by GPER1 rather than ERα and ERβ (224, 225). Furthermore, GPER1 activation by G1 agonist phosphorylates endothelial NOS, resulting in NO-mediated vasodilation in human endothelial cells (226). NO formation *via* G1 agonist is mediated by multiple pathways, such as the proto-oncogene tyrosine-protein kinase (c-Src), EGFR, PI3K, and ERK (226).

In conclusion, all three ERs plays protective roles in vascular disease (**Table 1**; **Figure 6**). ERα can reduce endothelial dysfunction, reduce RV hypertrophy, and increased antioxidation. ERβ decreases ROS production, inhibits inflammatory response, suppressed fibrosis, and decrease calcification. GPER1 reduces blood pressure, stimulates vasodilation, inhibits fibroblast proliferation, and decreases VSMC proliferation and migration.

### ERs in gastrointestinal disease

Gastrointestinal diseases are a collective manifestation of esophageal, gastric, and intestinal disorders such as hepatic and pancreatic diseases. Gastrointestinal diseases are highly associated with sex differences and female estrogen status (227–231), which indirectly indicates the potential role of hormones in gastrointestinal diseases. Considering the differential effects of ERs in different gastrointestinal diseases, we explored the multifaceted roles of estrogen and ERs in this section.

#### Esophageal diseases

Gastroesophageal reflux disease (GERD) and esophageal cancer (EC) are the primary esophageal diseases. GERD is a recurrent disease characterized by abnormal reflux at the gastroesophageal junction. EC, one of the most common malignant tumors in the gastrointestinal tract, is divided into two histological subtypes: esophageal adenocarcinoma (EAC) and esophageal squamous cell carcinoma (ESCC). EAC can arise from chronic GERD (232). The prevalence of GERD and EC in men is reportedly higher than in women. However, the risk of disease development rapidly increases in women who have undergone menopause (227). Notably, the incidence of ECA and ESCC is 6–10 times and 2–3 times lower, respectively, in women than in men (233). Estrogens play a protective role in GERD and EC. Estrogens exhibit anti-inflammatory functions and decrease esophageal tissue damage during GERD development (232). A study conducted in female rats showed that estrogen reduces esophageal tissue damage by binding to ERs (232). Boeckxstaens et al. also reported a close relationship between an increased prevalence of reflux esophagitis and reduced estrogen levels after menopause (234). A meta-analysis further demonstrated that estrogen could decrease the risk of EC and that hormone replacement therapy was negatively correlated with the risk of EC (235). Zhang et al. demonstrated the anti-proliferative function of estrogen in ESCC, and this effect was weakened by an ER antagonist (120). Estrogen may exert an anti-proliferative effect in ESCC cells by promoting the ER-calcium signaling pathway.

ERα and ERβ are highly expressed in ESCC cell lines. However, the expression level and role of ERα in ESCC remain controversial. Nozoe et al. demonstrated that ERα is expressed in the cytoplasm of 64.4% tumor tissue in immunohistochemistry analysis (122). Dong et al. suggested that ERα is related to improved survival and depth of tumor invasion (119). In contrast, Nozoe et al. reported poorer survival in association with ERα (122). A meta-analysis of 20 studies implied that higher ERα expression levels could be associated with poorer survival (121), highlighting the facilitating role of ERα in ESCC. The role of ERα in esophageal diseases warrants further exploration.

The role of ERβ in esophageal diseases also remains controversial. Wang et al. suggested that estrogen in the areas of EC high incidence is relatively lower, and the inhibition role of estrogen against tumor is mediated by the ERβ (236). Nozoe et al. demonstrated that the ERβ is expressed in nuclei of 28.8% tumor tissue (21/73) (122). They also found that the ERβ (–) is a poor prognostic factor, indicating a protective role in the EC. A previous study has suggested that ERβ in ESCC is typically associated with adverse outcomes (124), whereas estrogen and ERβ were shown to inhibit the cell proliferation in EC (123). Relatively fewer controversial results have been documented in GERD. The unanimity of these results indicates that estrogens and their receptors are highly associated with GERD risk, and hormone replacement therapy in women who have undergone menopause increases reflux symptoms (237).

Few studies have focused on GPER1 in esophageal diseases. One study revealed that GPER1 overexpression and upregulation of the cancer suppressor gene beclin-1 in ESCC is associated with poor prognosis (125). The authors emphasized that GPER1 promotes ESCC through increased cell proliferation and metastasis. p38 MAPK inhibitor may block the effects of GPER1, indicating that its tumor-promoting role in ESCC development is mediated by p38 MAPK.

Taken together, ERα and ERβ exhibits both inhibit and promote role in esophageal diseases (**Table 1**; **Figure 6**). Whereas, GPER1 may promote cell proliferation and metastasis in through p38 MAPK pathway.

#### Gastric diseases

Peptic ulcers (PUs) and gastric cancer (GCs) are the most common gastric diseases. PU results in gastric and duodenal ulcers with complications such as upper gastrointestinal bleeding and gastric outlet obstruction. PU is relatively rare during pregnancy, suggesting that estrogen may exert a protective effect. The risk of developing PUs is lower in women than in men (228, 229). Women also tend to develop ulcerative lesions after their menopause (238). The decrease in serum estrogen levels weakens the gastric mucosal defense mechanisms. Additionally, the antioxidant effects of estrogens directly eliminate free oxygen radicals, activate antioxidant enzymes, inhibit the production of superoxide, and decrease the formation of PUs (239). GC is the third most lethal malignant tumor. Similar to PU, the incidence of GC in men is reportedly higher than that in women. In contrast, the possibility of developing GC appears to be similar between women who have undergone menopause and men (240). Tokunaga et al. reported the protective function of estrogens against GC (241). Untreated male rats showed increased mobility rates compared to the castrated or estrogen-treated male rats with GC (242).

ERs play pivotal roles in the occurrence and development of GC. Although ERα and ERβ are expressed during GC, ERβ, but not ERα, is abundantly expressed in GCs (243). Some studies suggest over expressed ERα (244) while some suggest low expressed ERα in GC (245). ERα overexpression increases GC cell apoptosis and inhibits cell growth and proliferation (126). Another study reported that positive ERα expression in GC cells is related to poor prognosis in patients with GC, thereby highlighting its role in modulating cell proliferation, migration, and invasion by regulating the expression of p53, p21, p27, cyclin D1, and E-cadherin (127). In addition, ERα-36 tends to be highly expressed and is associated with lymph node metastasis in GC (128).

The expression level of ERβ in tumor tissue is lower than in healthy tissue (243, 245). ERβ is likely a protective factor against GC invasiveness (129). Suppression of ERβ can promote GC cell apoptosis by inducing autophagy (130). The malignant growth of GC tumor cells is mediated by overexpression of GRP78 and GRP94 (246, 247). In addition, Guo et al. detect the expression of ERβ1, ERβ2, and ERβ5 in GC tissues, and suggest that the expression of these three ERβ variants is found in most tumor tissues, and the expression of ERβ5 is significantly higher than that of ERβ1 and ERβ2. The high expression of ERβ5 in tumor tissues is associated with high tumor stage and lymph node metastasis, suggesting poor prognosis (131).

GPER1 expression levels are reportedly downregulated in GC tissues. The lower GPER1 expression tends to be negatively correlated with the survival of patients with GC (132). It also acts as a tumor suppressor by regulating the epithelial-mesenchymal transition pathway (132). In contrast, Zhang et al. detected higher GPER1 expression levels in GC than in normal tissues and suggested that this overexpression is associated with poor survival (133).In conclusion, ERα and GPER1 both shows controversial function in the progression of GC (**Table 1**; **Figure 6**). ERβ is a protective factor against GC invasiveness. Furthermore, ERα-36 is associated with lymph node metastasis in GC and high expression of ERβ5 in tumor tissues is associated with high tumor stage and lymph node metastasis, suggesting a poor prognosis.

#### Intestinal diseases

Irritable bowel syndrome (IBS), inflammatory bowel disease (IBD), and colon cancer (CC) are three major intestinal disorders. IBS is characterized by disorderly bowel movements and chronic abdominal pain. Some studies have highlighted the significant roles of estrogen and ERs in the pathogenesis of IBS. IBS (230) and CC (231) are more prevalent in women than in men, suggesting that estrogen participates in IBS and CC pathophysiology. IBS symptoms are reportedly related to hormones (248). IBD can be associated with the development of colorectal cancer, with men demonstrating a higher probability of developing colorectal cancer than women (249). Men also present a higher risk of developing colitis than women, suggesting a protective function of estrogen against colitis development (250). Collectively, estrogen, mediated by ERs, plays a promising and significant role in the development of intestinal disorders.

ERα expression is higher in IBS (138), and ERα-mediated cancer cell proliferation is considered a risk factor for CC (134). ERβ expression is lower in patients with IBD (251) and protects against colitis-associated neoplasia (250). ERβ upregulation indicates a better survival outcome, whereas its downregulation results in poor overall survival (135, 135). ERβ knockout results in greater damage in the gastrointestinal system of mice (136) and enhances tumor cell proliferation in both male and female (137). These findings underscore the protective effects of ERβ on CC. Higher GPER1 levels in patients with IBS (138) have been associated with pain severity (138). The GPER1-mediated estrogenic effects regulate visceral pain and gastrointestinal motility (139). The expression and inhibition of VEGFA by E2 can be mediated by a GPER1 dependent mechanism (231). Interestingly, when ERβ is absent in patients with CC, ERα and GPER1 are activated under anoxic conditions (231).

The expression of ERα-36 and ERα-46 in CC is higher than that in normal tissue. ERα-36 is associated with tumor stage and lymph node metastasis (252), suggesting that the two splice variants might be associated with CC development. Low ERβ2 expression in CC is likely associated with the occurrence of tumors in females (253, 254).

ERα and GPER1 tend to show harmful effects, while ERβ shows a protecting role in intestinal diseases (**Table 1**; **Figure 6**). More interestingly, Erα and GPER1 and will be active when ERβ is absent. Furthermore, splice variants of ERα-36, ERα-46, and ERβ2 may be involved in the CC development.

#### Liver disease

The liver is a hormone-sensitive organ, and its functions are influenced by estrogens (255). Common liver diseases include hepatitis, cirrhosis, liver abscesses, and liver cancer. Estrogens and their receptors play important roles in the liver (256). The role of estrogens in the liver tends to be controversial, as they both promote and inhibit chronic liver disease and carcinogenesis (257, 258). Many studies have verified the protective role of estrogen in ovariectomized rat models, whereas elevated estrogen levels have also been associated with chronic liver disease in some clinical studies (257). The role of estrogens in the development of hepatocellular carcinoma (HCC) is also controversial roles owing to its carcinogenic and protective effects on the liver (258).

In health liver tissue, men have higher ERα expression level than women, while no significant differences in ERβ expression. In addition, ERα:ERβ is higher in health men (143). ERα and ERβ are expressed in liver tissues and have promoted liver regeneration by orchestrating liver cell proliferation and differentiation (259). ERα is lower expressed in HCC, and is demonstrated to play an inhibiting role in tumor (260). ERα is demonstrated to up-regulate the expression of Protein Tyrosine phosphatase receptor Type O (PTPRO), which plays an inhibitory role in liver cancer (140). Similarly, Dai et al. reported that ERα gene is one of the tumor suppressor genes of HCC, and its methylation is associated with no microvascular infiltration and low histological grade (141). They believed that the protective role of ERα gene in HCC is related to its methylation. MicroRNA-221 can promote the proliferation of HCC cells by inhibiting the expression of ERα (142). Li et al. (261) reported that ERα expression is also down-regulated in female hepatitis B virus-associated HCC, and increased expression of microRNA-18 can inhibit ERα translation. They also found that up-regulated expression of p53 gene or mutation of p53 gene can inhibit ERα by regulating microRNA-18, thus weakening the anticancer effect of the ERα pathway. The overall survival rate and disease-free survival rate were higher in patients ERα positive (262). In men, liver ERα levels increase during the development of HCC (263). While in hepatitis C virus-associated HCC, ERα and ERβ expression is higher (143). The author points that higher ER expression is associated with higher NF-κB and cyclin D1 expression, which related to cell proliferation and invasion. ERα variants are expressed at higher levels in tumor tissues than in peritumoral tissues, further demonstrating the promoting role of ERα in HCC (255). A study demonstrated that ERα and its two splice variants are differentially expressed in the liver: healthy liver has high expression of ERα-36; patients with cirrhosis have moderate expression levels of ERα-66, ERα-46, and ERα-36; patients with HCC do not express ERα-66, but have a moderate ERα-46 expression and a high ERα-36 expression (258). These different expression patterns suggest that genetic variability may be vital in developing liver diseases.

ERβ has been reported to be related to lipid metabolism. ERβ activation by genistein (a natural phytoestrogen) improves hepatic lipid metabolism (264). ERβ is involved in the regulation of hepatic fibrosis (265). The anti-fibrotic effect of estrogen is mediated by ERβ but not by ERα or GPER1. ERβ-selective agonists ameliorate liver cirrhosis in rats by inhibiting HSC activation and proliferation of hepatic stellate cells (266). Estrogen inhibits the proliferation of HCC cells by inhibiting tumor-associated macrophages through ERβ (144). In addition, ERα and ERβ inhibit transcription and translation of peroxisome proliferator-activated receptor γ (PPARγ) gene in a ligand-dependent manner, and PPARγ can promote the proliferation of hepatocellular carcinoma cells (145).

The role of GPER1 in the liver is complex. The expression of GPER1 in HCC tissues is lower than that in non-HCC tissues (146). The development of liver tumors accelerates in HCC rat models with GPER1 knockout, and this effect is mediated by inflammatory responses, such as increased immune cell infiltration and IL-6 (146). These results demonstrated that GPER1 activity might be an effective strategy for preventing and treating HCC. Another study found that GPER1 is a protective factor in alleviating hepatocyte necrotic apoptosis in hepatic ischemia-reperfusion injury (147).

Taken together, ERβ and GPER1 show inhibiting role in the progress of liver cancer through reducing the fibrosis and immune response, while the role of ERα remains controversial (**Table 1**; **Figure 6**). When ERα works together with ERβ, they show an inhibitory role on PPARγ, which promoting the proliferation of hepatocellular carcinoma cells.

#### Pancreatic disease

The pancreas is an essential digestive organ in humans. The pancreas has endocrine and exocrine functions, mainly related to glucose metabolism. The external secretion of the pancreas is pancreatic juice, which is secreted from the pancreas and produces digestive enzymes that help digest fat. Islet cells produce insulin, which lowers blood sugar, and glucagon, which increases blood sugar. Pancreatic diseases include inflammatory diseases, pancreatic cysts, and other malignancies. When pancreatitis occurs, fat in the body is difficult to digest. Estrogens are highly associated with glucose metabolism (267). Furthermore, pancreatic ductal adenocarcinoma (PDAC) is the most common pancreatic cancer, and estrogen receptors may play an important role in its progression.

ERα is significantly expressed in pancreatic cancer tissues and is related to tumor size, distant metastasis, and poor prognosis of pancreatic cancer. ERα promotes cell proliferation, tumor growth, migration, and invasion by activating epithelial-mesenchymal transformation (148). ERα knockdown induced apoptosis and G0/G1 cell cycle arrest in pancreatic cancer cells. This study further demonstrated that ERα enhances the transcription of PAI1 (plasminogen activator inhibitor 1) and activates the MEK/ERK pathway, thereby promoting pancreatic cancer progression. These findings suggest that ERα may be a novel diagnostic and therapeutic target for pancreatic cancer (148). In most studies, ERα was detected in pancreatic tumors, while ERβ was absent, indicating that the role of ERβ in pancreatic cancer is unclear (268). The ERβ expression level in some cell lines is higher, and favorable prognostic features have been found with higher ERβ expression levels (269). A recent study showed that ERβ is mainly expressed in malignant cells and is considered a negative prognostic factor and possible therapeutic target for PDAC (270). Raloxifene has been demonstrated to reduce PDAC cells by disturbing the expression of ERβ and inhibiting the IL-6/gp130/STAT3 signaling pathway, indicating ERβ may carcinogenic role in pancreatic cancer (149).

High GPER1 expression in PDAC is associated with improved survival (150). Administering GPER1 agonist G1 reduced the tumor progression and prolonged survival of the treated mice (151). Activated GPER1 causes G1-S cell cycle arrest and corresponding decreases in p-RB and c-Myc. c-Myc, which is frequently overexpressed in cancer, is a transcription factor that stimulates proliferation and growth by activating many target genes. In addition, G1 significantly improved the efficacy of PD-1 targeted immunotherapy (151).

Collectively, ERα and ERβ show promoting role in pancreatic disease and may be the therapy target (**Table 1**; **Figure 6**). Activated GPER1 can inhibit the proliferation and growth of tumor cells and improve the efficacy of immunotherapy.

### ERs in urogenital tract disease

Urinary tract infections (UTIs) and urothelial carcinoma of the bladder (UCB) are the most common urogenital diseases. UTIs are bacterial infections, wherein 80% are caused by uropathogenic *Escherichia coli*. UCB is the most common bladder malignancy. Endogenous estrogen protects against UTIs (271–273). Postmenopausal women are more likely to develop recurrent UTIs (274). Many other studies have also emphasized the protective role of estrogens against UTIs (275, 276). In UCB, estrogen is likely related to its development. The protective roles of estrogens in UTIs and their carcinogenic role in UCB are mediated by ERs.

In UTI, ERα is related to the bacterial clearance functions. In ovariectomized mice, ERα agonist PPT treatment decreases the bacterial load in the kidney, whereas treatment with the ERα antagonist MPP (methyl-piperidino-pyrazole) impairs bacterial clearance. These results suggest that bacterial clearance in the kidney is mediated by ERα (271). Estrogen primarily stimulates urothelial cell proliferation through ERα and ERβ activation (277). High ERβ expression levels are detected in the epithelial cells of the urogenital tract, whereas the ERα expression level is much lower (278). However, significantly higher ERα expression levels are observed in bladder transitional cell carcinomas than in primary cells, but similar level of ERβ is found in that all of these urothelial cell (277, 279). Estrogen stimulates the increased ERα expression in male rats and induces urogenital carcinogenesis (152). ERβ also stimulates tumor cell growth (153) and is upregulated in UCB compared to that in the benign urothelium, suggesting an oncogenic function. Tamoxifen blocks cell proliferation (153). Teng et al. pointed that both ERα and ERβ contribute to estrogen-induced G1/S phase progression and cell proliferation in urothelial cells, and increased ERα expression may induce early cyclin D1 and cyclin E expression, thus resulting in dysregulated cell proliferation in bladder cancer cells (277).

Urothelial cells also express high GPER1 levels (154). A study suggested that E2 and the GPER1 agonist G1 would inhibit urothelial cell proliferation by mediating GPER1 activity. GPER1 overexpression also inhibited E2-induced cell proliferation, and siRNA-mediated decrease in GPER1 expression restored E2-induced cell proliferation (154). These results imply that the inhibitory effects of E2 on cell proliferation are mediated by GPER1. Mechanistically, GPER1 inhibits urothelial cell proliferation by decreasing cyclin D1 by downregulating activation of protein-1 signaling.

In conclusion, both ERα and ERβ stimulates urothelial cell proliferation and tumor cells growth, while GPER1 inhibit urothelial cell proliferation (**Table 1**; **Figure 6**). Furthermore, ERα shows function of bacterial clearance.

### ERs in neurodegenerative disease

Neurodegenerative diseases are characterized by decreased mitochondrial activity, oxidative phosphorylation, and increased ROS production in the CNS. Common neurodegenerative diseases include Alzheimer’s disease (AD) and Parkinson’s disease (PD). AD is a progressive neurodegenerative pathology characterized by extracellular amyloid plaques and intracellular neurofibrillary tangles, leading to neuronal dysfunction and cell death, negatively affecting cognition and memory in patients with AD (280). Postmenopausal women reportedly show a higher prevalence of AD and faster cognitive decline than men (281, 282). Some researchers have reported inhibition of aromatase (a crucial enzyme that converts androgens into estrogens) by altering β-amyloid deposition (283). Hormone replacement therapy decreases the risk of developing AD and decelerates declining cognitive function (284). Additionally, a meta-analysis demonstrated that hormone replacement therapy could benefit postmenopausal women with neurodegenerative diseases such as AD and PD (285). Several studies have shown the neuroprotective function of estrogens, which act as antioxidants, facilitating DNA repair, inducing growth factor expression, and regulating cerebral blood flow (282). Parkinson’s disease (PD) affects motor functions, which eventually progresses to cognitive impairment. Women with lower estrogen exposure are more likely to develop PD than those with higher estrogen exposure (286). E2, rather than androgens, has a protective effect on dopamine neurons (287). E2 also appears to have a protective effect on PD progression and treatment response (288).

ERα, ERβ, and GPER1 are expressed in cortical and hippocampal neural stem/progenitor cells. ERα is mainly associated with regulating reproductive functions, including the hypothalamus and the preoptic regions (156). ERα levels in female rats are often higher than those in male rats (155). Simultaneously, ERα is abundantly present in the prefrontal cortex of nonhuman primates. Ca^2+^ signaling plays a vital role in maintaining normal brain function. ERα- and ERβ-selective agonists can effectively activate rapid intracellular Ca^2+^ influx in neurons, leading to downstream MAPK signaling and ERK phosphorylation, thus playing a neuroprotective role (289). Hyperactivation of glutamate receptors can also induce excitotoxic neuronal damage, whereas ERα can prevent this damage *via* its estrogen signaling mechanism. Co-activation of ERα and IGF-IR may mediate neuroprotection *via* ERK and Akt phosphorylation in patients with AD (290).

ERβ is widely distributed and expressed in the hippocampus, cerebral cortex, lateral septa, and medial and basolateral amygdala. ERβ expression levels are higher than those of ERα in human and rat hippocampi (155). ERβ-knockdown mouse embryos showed lower cortical neuron migration and greater apoptosis than wild-type mice. Adult mice with ERβ knockdown showed abnormal morphology during brain development, including decreased neurons in cortical regions (291). Additionally, brain-derived neurotrophic factor (BDNF) regulates synaptic genesis and maturation. Activation of ERβ expression significantly increases the BDNF protein levels in postmenopausal mice, which plays a pivotal role in promoting the survival and differentiation of brain neurons (157, 158). ERα and ERβ distinctly contribute to neuroprotection against MPTP toxicity in mice (287). The striatum is one of the basal ganglia of the brain, and it regulates muscle tone and coordinates various fine and complex movements. Damaged striatum can impair its associated functions. ERα knockout mice are more sensitive to MPTP toxicity, whereas ERβ knockout mice have lower levels of striatal dopamine transporters.

GPER1 agonists reportedly increase dendritic spine density in the hippocampus. Hippocampal GPER1 also participates in estrogenic mediation of learning and memory *via* rapid signaling mechanisms (159). GPER is also involved in regulating hippocampal synaptic plasticity. BDNF expression induced by selective GPER1 activation promotes synaptic plasticity (292). Activation of the PI3K/Akt pathway can be correlated with GPER1 activation-conferred protection in AD models (160) and PD (161). GPER1 also mediated the E2 signaling pathway and exerted a neuroprotective action *via* regulating the PI3K/Akt and MAPK pathways in PD (292).

Like in the cardiovascular system, ERα, ERβ, and GPER1 all show neuroprotective role in nervous system (**Table 1**; **Figure 6**). ERα can stimulate Ca^2+^ influx and glutamate receptors in neurons and thus prevent the damage of neurodegenerative diseases. ERβ expression s increases the BDNF protein levels, thus promote the survival and differentiation of brain neurons. GPER1 may increase dendritic spine density in the hippocampus and synaptic plasticity.

### ERs in cutaneous melanoma

Melanoma is an aggressive tumor characterized by mutations in the oncogenes BRAF or NRAS, resulting in the overactivation of the MAPK/ERK and PI3K/Akt pathways (163). Current treatments for melanoma focus on mutated BRAF and its downstream pathways. Melanoma is traditionally considered a non-hormone-related cancer, while increasing evidence suggests a direct relationship between the sex hormones (especially estrogens) and melanoma (293). The prevalence and prognosis of melanoma are significantly associated with sex divergence. In melanoma, females possess a significant survival advantage (approximately 30%) over males (294). However, these advantages disappear in the postmenopausal period marked by reduced estrogen levels (295). Additional evidence has demonstrated that sex differences exist in tumor metastasis (296). Researchers have highlighted the vital role of estrogens in the differential manifestation of disorders between females and males.

ERα is the primary epidermal receptor in human tissues and is expressed at low levels in metastatic melanoma and pregnancy-associated melanoma (193, 293). Immunohistochemical observations from 38 patients with melanoma confirmed that ERα expression was absent malignant melanoma (297). Traditionally, ERα was thought to promote the growth of melanoma cells (298). Tian et al. demonstrated that ERα exhibited a pro-proliferative effect in melanoma by inducing the Akt and MAPK signaling pathways (162).

ERβ is predominant in all melanocytic lesions, including nevi and melanoma (163). de Giorgi et al. have found that the expression level of ERβ in melanoma is decreased when compared with health tissue (299). As the tumor progresses, the expression level of ERβ is decreasing, which is closely related to the occurrence and development of malignant melanoma (193, 300). Moreover, ERβ expression was reduced upon cellular exposure to ultraviolet (UV) light, resulting in thinner lesions with a prominent epidermis (293). In addition, the level of ERβ in women is significantly higher than that in men, which may be consistent with the better prognosis of female patients with malignant melanoma (301).These evidences imply that ERβ acts as a tumor suppressor in skin. Based on *in vitro* studies, ERβ ligands inhibit NRAS mutation and thus the proliferation of melanoma cells, demonstrating that ERβ activation might weaken melanoma development by preventing the PI3K/Akt pathway (163). These results suggest that ERβ agonists may be an effective treatment strategy for NRAS-mutant melanomas.

GPER1 controls melanin production and is expressed in melanoma cells of the skin (302). Specifically, GPER1 mediates melanocyte growth, differentiation, and function *via* intracellular cAMP-protein kinase and cAMP response element-binding protein (CREB) phosphorylation (293). GPER1 agonists inhibit melanoma cell proliferation, providing a protective effect against melanoma (302). Furthermore, GPER co-expressed with ERβ in melanoma tended to present a superior outcome, with lower Breslow thickness and mitotic rate as well as higher peritumoral lymphocyte infiltration (164). GPER1 signaling enhances melanoma cell sensitization to immunotherapy. Systemic treatment with a GPER1 agonist combined with immune checkpoint blockade dramatically increased survival in melanoma-bearing mice, with up to 50% of the mice exhibiting tumor clearance (165).

In conclusion, ERα and ERβ exhibits pro-proliferative effects in melanoma by activating the downstream pathway like Akt and MAPK signaling pathways (**Table 1**; **Figure 6**). GPER1 shows inhibiting role and can be a treatment target in the melanoma.

## Conclusions

Estrogens and their roles in ER signal transduction have become pivotal in developing various diseases, thereby making them potential therapeutic targets. ERs are expressed in various organs and regulate vital functions (203). We summarized the current knowledge on the role of ERs in health and their dysfunction, which leads to various diseases (**Table 1**, **Figure 6**). The diverse roles of ERs and their expression levels in various organs have been highlighted in our review. In some diseases like breast cancer, the expression condition and the role of ERs have been fully reported. ERα is normally considered to be a negative factor in the tumor growth, and ERβ inhibited the tumor by inhibiting the effects of ERα. In addition, the ERs show protective role in the bone, cardiovascular, and nervous systems. Nevertheless, the role of ERs in many diseases like gastric diseases remains unclear or controversial, indicating more studies is need to be conducted to further investigate the role of ERs in these diseases. In addition, different isoforms of ERs also exhibit significant roles in some disease like breast cancer, indicating more exploration on these isoforms needed to be conducted. Moreover, in the view of the different roles of ERs in the different organs or diseases, more comprehensive and specific should be done to reveal the synergistic or antagonistic action or more complex roles of ERs and their isoforms in different conditions. Furthermore, more new drugs targeting specific ERs should be developing in the therapy of relevant disease. Estrogen-mediated nuclear and cytoplasmic pathways are crucial in maintaining the physiology of multiple organ systems in both males and females. Further elucidation of the molecular mechanisms by which estrogen maintains homeostasis might provide deeper insights into the associated pathogenesis and clinical therapy pathways.

## Author contributions

BL and LOY data curation; PC writing original draft preparation; PC and BL writing—review and editing; LOY Supervision; PC visualization; All authors have read and agreed to the published version of the manuscript.

## Conflict of interest

The authors declare that the research was conducted in the absence of any commercial or financial relationships that could be construed as a potential conflict of interest.

## Publisher’s note

All claims expressed in this article are solely those of the authors and do not necessarily represent those of their affiliated organizations, or those of the publisher, the editors and the reviewers. Any product that may be evaluated in this article, or claim that may be made by its manufacturer, is not guaranteed or endorsed by the publisher.

## References

[1] Fuentes N Silveyra P. Estrogen receptor signaling mechanisms. Adv Protein Chem Struct Biol (2019) 116:135–70. doi: 10.1016/bs.apcsb.2019.01.001 PMC6533072 31036290

[2] Faltas CL LeBron KA Holz MK. Unconventional estrogen signaling in health and disease. Endocrinology (2020) 161(4):1–7. doi: 10.1210/endocr/bqaa030 PMC7101056 32128594

[3] Tang ZR Zhang R Lian ZX Deng SL Yu K. Estrogen-receptor expression and function in female reproductive disease. Cells (2019) 8(10):1123–38. doi: 10.3390/cells8101123 PMC6830311 31546660

[4] Flasch M Bueschl C Del Favero G Adam G Schuhmacher R Marko D. Elucidation of xenoestrogen metabolism by non-targeted, stable isotope-assisted mass spectrometry in breast cancer cells. Environ Int (2022) 158:106940. doi: 10.1016/j.envint.2021.106940 34673318

[5] Ohta K Chiba Y Kaise A Endo Y. Structure-activity relationship study of diphenylamine-based estrogen receptor (ER) antagonists. Bioorg Med Chem (2015) 23(4):861–7. doi: 10.1016/j.bmc.2014.12.022 25614118

[6] Kumar RS Goyal N. Estrogens as regulator of hematopoietic stem cell, immune cells and bone biology. Life Sci (2021) 269:119091. doi: 10.1016/j.lfs.2021.119091 33476629

[7] Russell JK Jones CK Newhouse PA. The role of estrogen in brain and cognitive aging. Neurotherapeutics (2019) 16(3):649–65. doi: 10.1007/s13311-019-00766-9 PMC6694379 31364065

[8] Shang Y. Molecular mechanisms of oestrogen and SERMs in endometrial carcinogenesis. Nat Rev Cancer (2006) 6(5):360–8. doi: 10.1038/nrc1879 16633364

[9] Bolger R Wiese TE Ervin K Nestich S Checovich W. Rapid screening of environmental chemicals for estrogen receptor binding capacity. Environ Health Perspect (1998) 106(9):551–7. doi: 10.1289/ehp.98106551 PMC1533147 9721254

[10] Delfosse V Maire AL Balaguer P Bourguet W. A structural perspective on nuclear receptors as targets of environmental compounds. Acta Pharmacol Sin (2015) 36(1):88–101. doi: 10.1038/aps.2014.133 25500867 PMC4571321

[11] O'Donnell L Robertson KM Jones ME Simpson ER. Estrogen and spermatogenesis. Endocr Rev (2001) 22(3):289–318. doi: 10.1210/er.22.3.289 11399746

[12] Jensen EV Suzuki T Numata M Smith S DeSombre ER. Estrogen-binding substances of target tissues. Steroids (1969) 13(4):417–27. doi: 10.1016/0039-128X(69)90053-1 5769572

[13] Walter P Green S Greene G Krust A Bornert JM Jeltsch JM. Cloning of the human estrogen receptor cDNA. Proc Natl Acad Sci (1985) 82(23):7889–93. doi: 10.1073/pnas.82.23.7889 PMC390875 3865204

[14] Kuiper G Enmark E Pelto-Huikko M Nilsson S Gustafsson JA. Cloning of a novel receptor expressed in rat prostate and ovary. Proc Natl Acad Sci (1996) 93(12):5925–30. doi: 10.1073/pnas.93.12.5925 PMC39164 8650195

[15] Filardo EJ Thomas P. Minireview: G protein-coupled estrogen receptor-1, GPER-1: its mechanism of action and role in female reproductive cancer, renal and vascular physiology. Endocrinology (2012) 153(7):2953–62. doi: 10.1210/en.2012-1061 PMC3380306 22495674

[16] Carmeci C Thompson DA Ring HZ Francke U Weigel RJ. Identification of a gene (GPR30) with homology to the G-protein-coupled receptor superfamily associated with estrogen receptor expression in breast cancer. Genomics (1997) 45(3):607–17. doi: 10.1006/geno.1997.4972 9367686

[17] Filardo EJ Quinn JA Bland KI Frackelton AR Jr. Estrogen-induced activation of erk-1 and erk-2 requires the G protein-coupled receptor homolog, GPR30, and occurs *via* trans-activation of the epidermal growth factor receptor through release of HB-EGF. Mol Endocrinol (2000) 14(10):1649–60. doi: 10.1210/mend.14.10.0532 11043579

[18] Maggiolini M Vivacqua A Fasanella G Recchia AG Sisci D Pezzi V. The G protein-coupled receptor GPR30 mediates c-fos up-regulation by 17beta-estradiol and phytoestrogens in breast cancer cells. J Biol Chem (2004) 279(26):27008–16. doi: 10.1074/jbc.M403588200 15090535

[19] Thomas P Pang Y Filardo EJ Dong J. Identity of an estrogen membrane receptor coupled to a G protein in human breast cancer cells. Endocrinology (2005) 146(2):624–32. doi: 10.1210/en.2004-1064 15539556

[20] Revankar CM Cimino DF Sklar LA Arterburn JB Prossnitz ER. A transmembrane intracellular estrogen receptor mediates rapid cell signaling. Science (2005) 307(5715):1625–30. doi: 10.1126/science.1106943 15705806

[21] Alexander SP Mathie A Peters JA. Guide to receptors and channels (GRAC), 5th edition. Br J Pharmacol (2011) 164 Suppl 1(Suppl 1):S1–324. doi: 10.1111/j.1476-5381.2011.01649_1.x 22040146 PMC3315626

[22] Luo J Liu D. Does GPER really function as a G protein-coupled estrogen receptor *in vivo* ? Front Endocrinol (Lausanne) (2020) 11:148. doi: 10.3389/fendo.2020.00148 32296387 PMC7137379

[23] Gosden JR Middleton PG Rout D. Localization of the human oestrogen receptor gene to chromosome 6q24–-q27 by *in situ* hybridization. Cytogenet Cell Genet (1986) 43(3-4):218–20. doi: 10.1159/000132325 3802924

[24] Enmark E Pelto-Huikko M Grandien K Lagercrantz S Lagercrantz J Fried G. Human estrogen receptor β-gene structure, chromosomal localization, and expression pattern. J Clin Endocrinol Metab (1997) 82(12):4258–65. doi: 10.1210/jc.82.12.4258 9398750

[25] Kumar R Zakharov MN Khan SH Miki R Jang H Toraldo G. The dynamic structure of the estrogen receptor. J Amino Acids (2011) 2011:812540. doi: 10.4061/2011/812540 22312471 PMC3268042

[26] Jia M Dahlman-Wright K Gustafsson J. Estrogen receptor alpha and beta in health and disease. Best Pract Res Clin Endocrinol Metab (2015) 29(4):557–68. doi: 10.1016/j.beem.2015.04.008 26303083

[27] Flouriot G Brand H Denger S Metivier R Kos M Reid G. Identification of a new isoform of the human estrogen receptor-alpha (hER-α) that is encoded by distinct transcripts and that is able to repress hER-α activation function 1. EMBO J (2000) 19(17):4688–700. doi: 10.1093/emboj/19.17.4688 PMC302047 10970861

[28] Wang Z Zhang X Shen P Loggie BW Chang Y Deuel TF. Identification, cloning, and expression of human estrogen receptor-alpha36, a novel variant of human estrogen receptor-alpha66. Biochem Biophys Res Commun (2005) 336(4):1023–7. doi: 10.1016/j.bbrc.2005.08.226 16165085

[29] Denger S Reid G Kos M Flouriot G Parsch D Brand H. ERalpha gene expression in human primary osteoblasts: evidence for the expression of two receptor proteins. Mol Endocrinol (2001) 15(12):2064–77. doi: 10.1210/mend.15.12.0741 11731609

[30] Shi L Dong B Li Z Lu Y Ouyang T Li J. Expression of ER-{alpha}36, a novel variant of estrogen receptor {alpha}, and resistance to tamoxifen treatment in breast cancer. J Clin Oncol (2009) 27(21):3423–9. doi: 10.1200/JCO.2008.17.2254 PMC2717750 19487384

[31] Moore JT McKee DD Slentz-Kesler K Moore LB Jones SA Horne EL. Cloning and characterization of human estrogen receptor β isoforms. Biochem Biophys Res Commun (1998) 247(1):75–8. doi: 10.1006/bbrc.1998.8738 9636657

[32] Warner M Fan X Strom A Wu W Gustafsson JÅ. 25 years of ERβ: a personal journey. J Mol Endocrinol (2021) 68(1):R1–r9. doi: 10.1530/JME-21-0121 34546964

[33] Swedenborg E Power KA Cai W Pongratz I Rüegg J. Regulation of estrogen receptor beta activity and implications in health and disease. Cell Mol Life Sci (2009) 66(24):3873–94. doi: 10.1007/s00018-009-0118-z PMC11115682 19669093

[34] Barton M Filardo EJ Lolait SJ Thomas P Maggiolini M Prossnitz ER. Twenty years of the G protein-coupled estrogen receptor GPER: Historical and personal perspectives. J Steroid Biochem Mol Biol (2018) 176:4–15. doi: 10.1016/j.jsbmb.2017.03.021 28347854 PMC5716468

[35] Prossnitz ER Barton M. Estrogen biology: new insights into GPER function and clinical opportunities. Mol Cell Endocrinol (2014) 389(1-2):71–83. doi: 10.1016/j.mce.2014.02.002 24530924 PMC4040308

[36] Olde B Leeb-Lundberg LM. GPR30/GPER1: searching for a role in estrogen physiology. Trends Endocrinol Metab (2009) 20(8):409–16. doi: 10.1016/j.tem.2009.04.006 19734054

[37] Mizukami Y. *In vivo* functions of GPR30/GPER-1, a membrane receptor for estrogen: from discovery to functions *in vivo* . Endocr J (2010) 57(2):101–7. doi: 10.1507/endocrj.K09E-332 19996532

[38] Klein SL Flanagan KL. Sex differences in immune responses. Nat Rev Immunol (2016) 16(10):626–38. doi: 10.1038/nri.2016.90 27546235

[39] Rothenberger NJ Somasundaram A Stabile LP. The role of the estrogen pathway in the tumor microenvironment. Int J Mol Sci (2018) 19(2):611. doi: 10.3390/ijms19020611 PMC5855833 29463044

[40] Kovats S. Estrogen receptors regulate innate immune cells and signaling pathways. Cell Immunol (2015) 294(2):63–9. doi: 10.1016/j.cellimm.2015.01.018 PMC4380804 25682174

[41] Pelekanou V Kampa M Kiagiadaki F Deli A Theodoropoulos P Agrogiannis G. Estrogen anti-inflammatory activity on human monocytes is mediated through cross-talk between estrogen receptor ERα36 and GPR30/GPER1. J Leukoc Biol (2016) 99(2):333–47. doi: 10.1189/jlb.3A0914-430RR 26394816

[42] Millas I Duarte Barros M. Estrogen receptors and their roles in the immune and respiratory systems. Anat Rec (Hoboken) (2021) 304(6):1185–93. doi: 10.1002/ar.24612 33856123

[43] Szostakowska M Trębińska-Stryjewska A Grzybowska EA Fabisiewicz A. Resistance to endocrine therapy in breast cancer: molecular mechanisms and future goals. Breast Cancer Res Treat (2019) 173(3):489–97. doi: 10.1007/s10549-018-5023-4 PMC6394602 30382472

[44] Huang B Omoto Y Iwase H Yamashita H Toyama T Coombes RC. Differential expression of estrogen receptor α, β1, and β2 in lobular and ductal breast cancer. Proc Natl Acad Sci U S A (2014) 111(5):1933–8. doi: 10.1073/pnas.1323719111 PMC3918808 24449868

[45] Couse JF Korach KS. Estrogen receptor null mice: what have we learned and where will they lead us? Endocr Rev (1999) 20(3):358–417. doi: 10.1210/edrv.20.3.0370 10368776

[46] Anbalagan M Rowan BG. Estrogen receptor alpha phosphorylation and its functional impact in human breast cancer. Mol Cell Endocrinol (2015) 418 Pt 3:264–72. doi: 10.1016/j.mce.2015.01.016 25597633

[47] Droog M Beelen K Linn S Zwart W. Tamoxifen resistance: from bench to bedside. Eur J Pharmacol (2013) 717(1-3):47–57. doi: 10.1016/j.ejphar.2012.11.071 23545365

[48] Wang Q Jiang J Ying G Xie XQ Zhang X Xu W. Tamoxifen enhances stemness and promotes metastasis of ERα36(+) breast cancer by upregulating ALDH1A1 in cancer cells. Cell Res (2018) 28(3):336–58. doi: 10.1038/cr.2018.15 PMC5835774 29393296

[49] Zhao C Lam EW Sunters A Enmark E De Bella MT Coombes RC. Expression of estrogen receptor β isoforms in normal breast epithelial cells and breast cancer: regulation by methylation. Oncogene (2003) 22(48):7600–6. doi: 10.1038/sj.onc.1207100 14576822

[50] Treeck O Juhasz-Boess I Lattrich C Horn F Goerse R Ortmann O. Effects of exon-deleted estrogen receptor β transcript variants on growth, apoptosis and gene expression of human breast cancer cell lines. Breast Cancer Res Treat (2008) 110(3):507–20. doi: 10.1007/s10549-007-9749-7 17876701

[51] Omoto Y Inoue S Ogawa S Toyama T Yamashita H Muramatsu M. Clinical value of the wild-type estrogen receptor β expression in breast cancer. Cancer Lett (2001) 163(2):207–12. doi: 10.1016/S0304-3835(00)00680-7 11165756

[52] Hopp TA Weiss HL Parra IS Cui Y Osborne CK Fuqua SA. Low levels of estrogen receptor β protein predict resistance to tamoxifen therapy in breast cancer. Clin Cancer Res (2004) 10(22):7490–9. doi: 10.1158/1078-0432.CCR-04-1114 15569979

[53] Cotrim C Fabris V Doria ML Lindberg K Gustafsson JÅ Amado F. Estrogen receptor beta growth-inhibitory effects are repressed through activation of MAPK and PI3K signalling in mammary epithelial and breast cancer cells. Oncogene (2013) 32(19):2390–402. doi: 10.1038/onc.2012.261 22751110

[54] Speirs V Malone C Walton DS Kerin MJ Atkin SL. Increased expression of estrogen receptor β mRNA in tamoxifen-resistant breast cancer patients. Cancer Res (1999) 59(21):5421–4. 10554009

[55] Markey G Cullen R Diggin P Hill AD Mc Dermott EW O'Higgins NJ. Estrogen receptor-β mRNA is associated with adverse outcome in patients with breast cancer. Tumor Biol (2009) 30(4):171–5. doi: 10.1159/000236409 19738412

[56] Neill O Davies MP Shaaban AM Innes H Torevell A Sibson DR. Wild-type oestrogen receptor beta (ERbetaI) mRNA and protein expression in tamoxifen-treated post-menopausal breast cancers. Br J Cancer (2004) 91(9):1694–702. doi: 10.1038/sj.bjc.6602183 PMC2409946 15477865

[57] Shaaban AM Green AR Karthik S Alizadeh Y Hughes TA Harkins L. Nuclear and cytoplasmic expression of ERbeta1, ERbeta2, and ERbeta5 identifies distinct prognostic outcome for breast cancer patients. Clin Cancer Res (2008) 14(16):5228–35. doi: 10.1158/1078-0432.CCR-07-4528 18698041

[58] Ignatov T Weißenborn C Poehlmann A Lemke A Semczuk A Roessner A. GPER-1 expression decreases during breast cancer tumorigenesis. Cancer Invest (2013) 31(5):309–15. doi: 10.3109/07357907.2013.789901 23688258

[59] Treeck O Schüler-Toprak S Ortmann O. Estrogen actions in triple-negative breast cancer. Cells (2020) 9(11):2358–73. doi: 10.3390/cells9112358 PMC7692567 33114740

[60] Ignatov T Treeck O Kalinski T Ortmann O Ignatov A. GPER-1 expression is associated with a decreased response rate to primary tamoxifen therapy of breast cancer patients. Arch Gynecol Obstet (2020) 301(2):565–71. doi: 10.1007/s00404-019-05384-6 31900584

[61] Liu L Liu S Luo H Chen C Zhang X He L. GPR30-mediated HMGB1 upregulation in CAFs induces autophagy and tamoxifen resistance in ERα-positive breast cancer cells. Aging (Albany NY) (2021) 13(12):16178–97. doi: 10.18632/aging.203145 PMC8266353 34182538

[62] Liang S Chen Z Jiang G Zhou Y Liu Q Su Q. Activation of GPER suppresses migration and angiogenesis of triple negative breast cancer *via* inhibition of NF-κB/IL-6 signals. Cancer Lett (2017) 386:12–23. doi: 10.1016/j.canlet.2016.11.003 27836733

[63] Zhao Y Gong P Chen Y Nwachukwu JC Srinivasan S Ko C. Dual suppression of estrogenic and inflammatory activities for targeting of endometriosis. Sci Transl Med (2015) 7(271):271ra9. doi: 10.1126/scitranslmed.3010626 PMC4790140 25609169

[64] Han SJ Jung SY Wu SP Hawkins SM Park MJ Kyo S. Estrogen receptor β modulates apoptosis complexes and the inflammasome to drive the pathogenesis of endometriosis. Cell (2015) 163(4):960–74. doi: 10.1016/j.cell.2015.10.034 PMC4640214 26544941

[65] Imesch P Samartzis EP Dedes KJ Fink D Fedier A. Histone deacetylase inhibitors down-regulate G-protein-coupled estrogen receptor and the GPER-antagonist G-15 inhibits proliferation in endometriotic cells. Fertil Steril (2013) 100(3):770–6. doi: 10.1016/j.fertnstert.2013.05.008 23755949

[66] Langdon SP Herrington CS Hollis RL Gourley C. Estrogen signaling and its potential as a target for therapy in ovarian cancer. Cancers (Basel) (2020) 12(6):1647–64. doi: 10.3390/cancers12061647 PMC7352420 32580290

[67] Bossard C Busson M Vindrieux D Gaudin F Machelon V Brigitte M. Potential role of estrogen receptor beta as a tumor suppressor of epithelial ovarian cancer. PloS One (2012) 7(9):e44787. doi: 10.1371/journal.pone.0044787 22970307 PMC3435304

[68] Chan KKL Siu MKY Jiang YX Wang JJ Wang Y Leung THY. Differential expression of estrogen receptor subtypes and variants in ovarian cancer: effects on cell invasion, proliferation and prognosis. BMC Cancer (2017) 17(1):606. doi: 10.1186/s12885-017-3601-1 28859612 PMC5579953

[69] Ciucci A Zannoni GF Travaglia D Petrillo M Scambia G Gallo D. Prognostic significance of the estrogen receptor beta (ERβ) isoforms ERβ1, ERβ2, and ERβ5 in advanced serous ovarian cancer. Gynecol Oncol (2014) 132(2):351–9. doi: 10.1016/j.ygyno.2013.12.027 24378878

[70] Ignatov T Modl S Thulig M Weißenborn C Treeck O Ortmann O. GPER-1 acts as a tumor suppressor in ovarian cancer. J Ovarian Res (2013) 6(1):51. doi: 10.1186/1757-2215-6-51 23849542 PMC3723961

[71] Smith HO Arias-Pulido H Kuo DY Howard T Qualls CR Lee SJ. GPR30 predicts poor survival for ovarian cancer. Gynecol Oncol (2009) 114(3):465–71. doi: 10.1016/j.ygyno.2009.05.015 PMC2921775 19501895

[72] Zhu CX Xiong W Wang ML Yang J Shi HJ Chen HQ. Nuclear G protein-coupled oestrogen receptor (GPR30) predicts poor survival in patients with ovarian cancer. J Int Med Res (2018) 46(2):723–31. doi: 10.1177/0300060517717625 PMC5971494 29239277

[73] Yan Y Jiang X Zhao Y Wen H Liu G. Role of GPER on proliferation, migration and invasion in ligand-independent manner in human ovarian cancer cell line SKOV3. Cell Biochem Funct (2015) 33(8):552–9. doi: 10.1002/cbf.3154 26526233

[74] Albanito L Madeo A Lappano R Vivacqua A Rago V Carpino A. G Protein-coupled receptor 30 (GPR30) mediates gene expression changes and growth response to 17beta-estradiol and selective GPR30 ligand G-1 in ovarian cancer cells. Cancer Res (2007) 67(4):1859–66. doi: 10.1158/0008-5472.CAN-06-2909 17308128

[75] Marengo SR Chung LW. An orthotopic model for the study of growth factors in the ventral prostate of the rat: effects of epidermal growth factor and basic fibroblast growth factor. J Androl (1994) 15(4):277–86. 7982795

[76] Tørring N Vinter-Jensen L Pedersen SB Sørensen FB Flyvbjerg A Nexø E. Systemic administration of insulin-like growth factor I (IGF-I) causes growth of the rat prostate. J Urol (1997) 158(1):222–7. doi: 10.1097/00005392-199707000-00075 9186364

[77] Bonkhoff H. Estrogen receptor signaling in prostate cancer: Implications for carcinogenesis and tumor progression. Prostate (2018) 78(1):2–10. doi: 10.1002/pros.23446 29094395

[78] McPherson SJ Hussain S Balanathan P Hedwards SL Niranjan B Grant M. Estrogen receptor–β activated apoptosis in benign hyperplasia and cancer of the prostate is androgen independent and TNFα mediated. Proc Natl Acad Sci (2010) 107(7):3123–8. doi: 10.1073/pnas.0905524107 PMC2840300 20133657

[79] Ramírez-de-Arellano A Pereira-Suárez AL Rico-Fuentes C López-Pulido EI Villegas-Pineda JC Sierra-Diaz E. Distribution and effects of estrogen receptors in prostate cancer: Associated molecular mechanisms. Front Endocrinol (2022) 12. doi: 10.3389/fendo.2021.811578 PMC8786725 35087479

[80] Chan QK Lam HM Ng CF Lee AY Chan ES Ng HK. Activation of GPR30 inhibits the growth of prostate cancer cells through sustained activation of Erk1/2, c-jun/c-fos-dependent upregulation of p21, and induction of G(2) cell-cycle arrest. Cell Death Differ (2010) 17(9):1511–23. doi: 10.1038/cdd.2010.20 PMC2897932 20203690

[81] Liu S Le May C Wong WP Ward RD Clegg DJ Marcelli M. Importance of extranuclear estrogen receptor-alpha and membrane G protein-coupled estrogen receptor in pancreatic islet survival. Diabetes (2009) 58(10):2292–302. doi: 10.2337/db09-0257 PMC2750222 19587358

[82] Bord S Horner A Beavan S Compston J. Estrogen receptors alpha and beta are differentially expressed in developing human bone. J Clin Endocrinol Metab (2001) 86(5):2309–14. doi: 10.1210/jcem.86.5.7513 11344243

[83] Sims NA Dupont S Krust A Clement-Lacroix P Minet D Resche-Rigon M. Deletion of estrogen receptors reveals a regulatory role for estrogen receptors-beta in bone remodeling in females but not in males. Bone (2002) 30(1):18–25. doi: 10.1016/S8756-3282(01)00643-3 11792560

[84] Windahl SH Vidal O Andersson G Gustafsson JA Ohlsson C. Increased cortical bone mineral content but unchanged trabecular bone mineral density in female ERbeta(-/-) mice. J Clin Invest (1999) 104(7):895–901. doi: 10.1172/JCI6730 10510330 PMC408552

[85] Windahl SH Hollberg K Vidal O Gustafsson JA Ohlsson C Andersson G. Female estrogen receptor beta-/- mice are partially protected against age-related trabecular bone loss. J Bone Miner Res (2001) 16(8):1388–98. doi: 10.1359/jbmr.2001.16.8.1388 11499861

[86] Heino TJ Chagin AS Sävendahl L. The novel estrogen receptor G-protein-coupled receptor 30 is expressed in human bone. J Endocrinol (2008) 197(2):R1–6. doi: 10.1677/JOE-07-0629 18434348

[87] Mårtensson UE Salehi SA Windahl S Gomez MF Swärd K Daszkiewicz-Nilsson J. Deletion of the G protein-coupled receptor 30 impairs glucose tolerance, reduces bone growth, increases blood pressure, and eliminates estradiol-stimulated insulin release in female mice. Endocrinology (2009) 150(2):687–98. doi: 10.1210/en.2008-0623 18845638

[88] Ford J Hajibeigi A Long M Hahner L Gore C Hsieh JT. GPR30 deficiency causes increased bone mass, mineralization, and growth plate proliferative activity in male mice. J Bone Miner Res (2011) 26(2):298–307. doi: 10.1002/jbmr.209 20734455 PMC3179349

[89] Taylor AH Al-Azzawi F. Immunolocalisation of oestrogen receptor beta in human tissues. J Mol Endocrinol (2000) 24(1):145–55. doi: 10.1677/jme.0.0240145 10657006

[90] He M Yu W Chang C Miyamoto H Liu X Jiang K. Estrogen receptor α promotes lung cancer cell invasion *via* increase of and cross-talk with infiltrated macrophages through the CCL2/CCR2/MMP9 and CXCL12/CXCR4 signaling pathways. Mol Oncol (2020) 14(8):1779–99. doi: 10.1002/1878-0261.12701 PMC7400793 32356397

[91] Liu S Hu C Li M An J Zhou W Guo J. Estrogen receptor beta promotes lung cancer invasion *via* increasing CXCR4 expression. Cell Death Dis (2022) 13(1):70. doi: 10.1038/s41419-022-04514-4 35064116 PMC8782891

[92] Liu C Liao Y Fan S Tang H Jiang Z Zhou B. G Protein-coupled estrogen receptor (GPER) mediates NSCLC progression induced by 17β-estradiol (E2) and selective agonist G1. Med Oncol (2015) 32(4):104. doi: 10.1007/s12032-015-0558-2 25744245

[93] Wright AF Ewart MA Mair K Nilsen M Dempsie Y Loughlin L. Oestrogen receptor alpha in pulmonary hypertension. Cardiovasc Res (2015) 106(2):206–16. doi: 10.1093/cvr/cvv106 PMC4615797 25765937

[94] Ortmann J Veit M Zingg S Di Santo S Traupe T Yang Z. Estrogen receptor-α but not -β or GPER inhibits high glucose-induced human VSMC proliferation: potential role of ROS and ERK. J Clin Endocrinol Metab (2011) 96(1):220–8. doi: 10.1210/jc.2010-0943 PMC3038487 20962025

[95] Schubert C Raparelli V Westphal C Dworatzek E Petrov G Kararigas G. Reduction of apoptosis and preservation of mitochondrial integrity under ischemia/reperfusion injury is mediated by estrogen receptor β. Biol Sex Differ (2016) 7:53. doi: 10.1186/s13293-016-0104-8 27688871 PMC5035458

[96] Booth EA Obeid NR Lucchesi BR. Activation of estrogen receptor-alpha protects the in vivo rabbit heart from ischemia-reperfusion injury. Am J Physiol Heart Circ Physiol (2005) 289(5):H2039–47. doi: 10.1152/ajpheart.00479.2005 15994857

[97] Zhai P Eurell TE Cooke PS Lubahn DB Gross DR. Myocardial ischemia-reperfusion injury in estrogen receptor-alpha knockout and wild-type mice. Am J Physiol Heart Circ Physiol (2000) 278(5):H1640–7. doi: 10.1152/ajpheart.2000.278.5.H1640 10775144

[98] Westphal C Schubert C Prelle K Penkalla A Fliegner D Petrov G. Effects of estrogen, an ERα agonist and raloxifene on pressure overload induced cardiac hypertrophy. PloS One (2012) 7(12):e50802. doi: 10.1371/journal.pone.0050802 23227210 PMC3515519

[99] Mahmoodzadeh S Eder S Nordmeyer J Ehler E Huber O Martus P. Estrogen receptor alpha up-regulation and redistribution in human heart failure. FASEB J (2006) 20(7):926–34. doi: 10.1096/fj.05-5148com 16675850

[100] Frump AL Goss KN Vayl A Albrecht M Fisher A Tursunova R. Estradiol improves right ventricular function in rats with severe angioproliferative pulmonary hypertension: effects of endogenous and exogenous sex hormones. Am J Physiol Lung Cell Mol Physiol (2015) 308(9):L873–90. doi: 10.1152/ajplung.00006.2015 PMC4421786 25713318

[101] Billon-Galés A Fontaine C Douin-Echinard V Delpy L Berges H Calippe B. Endothelial estrogen receptor-alpha plays a crucial role in the atheroprotective action of 17beta-estradiol in low-density lipoprotein receptor-deficient mice. Circulation (2009) 120(25):2567–76. doi: 10.1161/CIRCULATIONAHA.109.898445 19996016

[102] Christian RC Liu PY Harrington S Ruan M Miller VM Fitzpatrick LA. Intimal estrogen receptor (ER)beta, but not ERalpha expression, is correlated with coronary calcification and atherosclerosis in pre- and postmenopausal women. J Clin Endocrinol Metab (2006) 91(7):2713–20. doi: 10.1210/jc.2005-2672 16608893

[103] Pelzer T Loza PA Hu K Bayer B Dienesch C Calvillo L. Increased mortality and aggravation of heart failure in estrogen receptor-beta knockout mice after myocardial infarction. Circulation (2005) 111(12):1492–8. doi: 10.1161/01.CIR.0000159262.18512.46 15781739

[104] Iorga A Umar S Ruffenach G Aryan L Li J Sharma S. Estrogen rescues heart failure through estrogen receptor beta activation. Biol Sex Differ (2018) 9(1):48. doi: 10.1186/s13293-018-0206-6 30376877 PMC6208048

[105] Kararigas G Fliegner D Gustafsson JÅ Regitz-Zagrosek V. Role of the estrogen/estrogen-receptor-beta axis in the genomic response to pressure overload-induced hypertrophy. Physiol Genomics (2011) 43(8):438–46. doi: 10.1152/physiolgenomics.00199.2010 21325064

[106] Liu H Pedram A Kim JK. Oestrogen prevents cardiomyocyte apoptosis by suppressing p38α-mediated activation of p53 and by down-regulating p53 inhibition on p38β. Cardiovasc Res (2011) 89(1):119–28. doi: 10.1093/cvr/cvq265 PMC3002868 20724307

[107] Shen D Tian L Shen T Sun H Liu P. Alpha-lipoic acid protects human aortic endothelial cells against H2O2-induced injury and inhibits atherosclerosis in ovariectomized low density lipoprotein receptor knock-out mice. Cell Physiol Biochem (2018) 47(6):2261–77. doi: 10.1159/000491537 29975924

[108] Umar S Iorga A Matori H Nadadur RD Li J Maltese F. Estrogen rescues preexisting severe pulmonary hypertension in rats. Am J Respir Crit Care Med (2011) 184(6):715–23. doi: 10.1164/rccm.201101-0078OC PMC3208600 21700911

[109] Lahm T Crisostomo PR Markel TA Wang M Wang Y Tan J. Selective estrogen receptor-alpha and estrogen receptor-beta agonists rapidly decrease pulmonary artery vasoconstriction by a nitric oxide-dependent mechanism. Am J Physiol Regul Integr Comp Physiol (2008) 295(5):R1486–93. doi: 10.1152/ajpregu.90667.2008 PMC2584849 18832085

[110] Pedram A Razandi M Narayanan R Levin ER. Estrogen receptor beta signals to inhibition of cardiac fibrosis. Mol Cell Endocrinol (2016) 434:57–68. doi: 10.1016/j.mce.2016.06.018 27321970

[111] Farhat MY Chen MF Bhatti T Iqbal A Cathapermal S Ramwell PW. Protection by oestradiol against the development of cardiovascular changes associated with monocrotaline pulmonary hypertension in rats. Br J Pharmacol (1993) 110(2):719–23. doi: 10.1111/j.1476-5381.1993.tb13871.x PMC2175952 8242243

[112] Delgado NTB Rouver WDN Freitas-Lima LC Vieira-Alves I Lemos VS Dos Santos RL. Sex differences in the vasodilation mediated by G protein-coupled estrogen receptor (GPER) in hypertensive rats. Front Physiol (2021) 12:659291. doi: 10.3389/fphys.2021.659291 34393807 PMC8359777

[113] Bopassa JC Eghbali M Toro L Stefani E. A novel estrogen receptor GPER inhibits mitochondria permeability transition pore opening and protects the heart against ischemia-reperfusion injury. Am J Physiol Heart Circ Physiol (2010) 298(1):H16–23. doi: 10.1152/ajpheart.00588.2009 PMC2806134 19880667

[114] Feng Y Madungwe NB da Cruz Junho CV Bopassa JC. Activation of G protein-coupled oestrogen receptor 1 at the onset of reperfusion protects the myocardium against ischemia/reperfusion injury by reducing mitochondrial dysfunction and mitophagy. Br J Pharmacol (2017) 174(23):4329–44. doi: 10.1111/bph.14033 PMC5715577 28906548

[115] Wang H Sun X Lin MS Ferrario CM Van Remmen H Groban L. G Protein-coupled estrogen receptor (GPER) deficiency induces cardiac remodeling through oxidative stress. Transl Res (2018) 199:39–51. doi: 10.1016/j.trsl.2018.04.005 29758174 PMC6151279

[116] Da Silva JS Sun X Ahmad S Wang H Sudo RT Varagic J. G-Protein-Coupled estrogen receptor agonist G1 improves diastolic function and attenuates cardiac renin-angiotensin system activation in estrogen-deficient hypertensive rats. J Cardiovasc Pharmacol (2019) 74(5):443–52. doi: 10.1097/FJC.0000000000000721 31361702

[117] Kang S Liu Y Sun D Zhou C Liu A Xu C. Chronic activation of the G protein-coupled receptor 30 with agonist G-1 attenuates heart failure. PloS One (2012) 7(10):e48185. doi: 10.1371/journal.pone.0048185 23110207 PMC3482180

[118] Wang H Zhao Z Lin M Groban L. Activation of GPR30 inhibits cardiac fibroblast proliferation. Mol Cell Biochem (2015) 405(1-2):135–48. doi: 10.1007/s11010-015-2405-3 PMC4449333 25893735

[119] Dong J Jiang SW Niu Y Chen L Liu S Ma T. Expression of estrogen receptor α and β in esophageal squamous cell carcinoma. Oncol Rep (2013) 30(6):2771–6. doi: 10.3892/or.2013.2770 24101172

[120] Zhang Z He Q Fu S Zheng Z. Estrogen receptors in regulating cell proliferation of esophageal squamous cell carcinoma: Involvement of intracellular Ca(2+) signaling. Pathol Oncol Res (2017) 23(2):329–34. doi: 10.1007/s12253-016-0105-2 27595756

[121] Zhang D Ku J Yi Y Zhang J Liu R Tang N. The prognostic values of estrogen receptor alpha and beta in patients with gastroesophageal cancer: A meta-analysis. Med (Baltimore) (2019) 98(46):e17954. doi: 10.1097/MD.0000000000017954 PMC6867741 31725654

[122] Nozoe T Oyama T Takenoyama M Hanagiri T Sugio K Yasumoto K. Significance of immunohistochemical expression of estrogen receptors alpha and beta in squamous cell carcinoma of the esophagus. Clin Cancer Res (2007) 13(14):4046–50. doi: 10.1158/1078-0432.CCR-07-0449 17634528

[123] Ueo H Matsuoka H Sugimachi K Kuwano H Mori M Akiyoshi T. Inhibitory effects of estrogen on the growth of a human esophageal carcinoma cell line. Cancer Res (1990) 50(22):7212–5. 2224855

[124] Zuguchi M Miki Y Onodera Y Fujishima F Takeyama D Okamoto H. Estrogen receptor α and β in esophageal squamous cell carcinoma. Cancer Sci (2012) 103(7):1348–55. doi: 10.1111/j.1349-7006.2012.02288.x PMC7659275 22463081

[125] Yang S Deng L Lai Y Liu Z. Over expression of GPR30, indicating poor prognosis and promoting proliferation, upregulates beclin-1 expression *via* p38MAPK signaling in esophageal squamous cell carcinoma progression. Int J Clin Exp Pathol (2018) 11(7):3426–35. PMC6962856 31949720

[126] Zhou J Teng R Xu C Wang Q Guo J Xu C. Overexpression of ERα inhibits proliferation and invasion of MKN28 gastric cancer cells by suppressing β-catenin. Oncol Rep (2013) 30(4):1622–30. doi: 10.3892/or.2013.2610 23843035

[127] Tang W Liu R Yan Y Pan X Wang M Han X. Expression of estrogen receptors and androgen receptor and their clinical significance in gastric cancer. Oncotarget (2017) 8(25):40765–77. doi: 10.18632/oncotarget.16582 PMC5522298 28388558

[128] Deng H Huang X Fan J Wang L Xia Q Yang X. A variant of estrogen receptor-alpha, ER-alpha36 is expressed in human gastric cancer and is highly correlated with lymph node metastasis. Oncol Rep (2010) 24(1):171–6. PMC3380086 20514458

[129] Ryu WS Kim JH Jang YJ Park SS Um JW Park SH. Expression of estrogen receptors in gastric cancer and their clinical significance. J Surg Oncol (2012) 106(4):456–61. doi: 10.1002/jso.23097 22422271

[130] Zhou F Jin J Zhou L Wu L Cao Y Yan H. Suppression of estrogen receptor-beta promotes gastric cancer cell apoptosis with induction of autophagy. Am J Transl Res (2020) 12(8):4397–409. PMC7476128 32913514

[131] Guo JL Xu CY Jiang ZN Dong MJ Xie SD Shen JG. Estrogen receptor beta variants mRNA expressions in gastric cancer tissues and association with clinicopathologic parameters. Hepatogastroenterology (2010) 57(104):1584–8. 21443125

[132] Tian S Zhan N Li R Dong W. Downregulation of G protein-coupled estrogen receptor (GPER) is associated with reduced prognosis in patients with gastric cancer. Med Sci Monit (2019) 25:3115–26. doi: 10.12659/MSM.913634 PMC6503750 31028714

[133] Zheng S Yang L Dai Y Jiang L Wei Y Wen H. Screening and survival analysis of hub genes in gastric cancer based on bioinformatics. J Comput Biol (2019) 26(11):1316–25. doi: 10.1089/cmb.2019.0119 31233344

[134] Chen J Iverson D. Estrogen in obesity-associated colon cancer: friend or foe? protecting postmenopausal women but promoting late-stage colon cancer. Cancer Causes Control (2012) 23(11):1767–73. doi: 10.1007/s10552-012-0066-z 23011535

[135] Konstantinopoulos PA Kominea A Vandoros G Sykiotis GP Andricopoulos P Varakis I. Oestrogen receptor beta (ERβ) is abundantly expressed in normal colonic mucosa, but declines in colon adenocarcinoma paralleling the tumour's dedifferentiation. Eur J Cancer (2003) 39(9):1251–8. doi: 10.1016/S0959-8049(03)00239-9 12763213

[136] Saleiro D Murillo G Benya RV Bissonnette M Hart J Mehta RG. Estrogen receptor-β protects against colitis-associated neoplasia in mice. Int J Cancer (2012) 131(11):2553–61. doi: 10.1002/ijc.27578 PMC3404195 22488198

[137] Hases L Indukuri R Birgersson M Nguyen-Vu T Lozano R Saxena A. Intestinal estrogen receptor beta suppresses colon inflammation and tumorigenesis in both sexes. Cancer Lett (2020) 492:54–62. doi: 10.1016/j.canlet.2020.06.021 32711097

[138] Qin B Dong L Guo X Jiang J He Y Wang X. Expression of G protein-coupled estrogen receptor in irritable bowel syndrome and its clinical significance. Int J Clin Exp Pathol (2014) 7(5):2238–46. PMC4069936 24966932

[139] Zielińska M Fichna J Bashashati M Habibi S Sibaev A Timmermans JP. G Protein-coupled estrogen receptor and estrogen receptor ligands regulate colonic motility and visceral pain. Neurogastroenterol Motil (2017) 29(7):1–11. doi: 10.1111/nmo.13025 28191706

[140] Hou J Xu J Jiang R Wang Y Chen C Deng L. Estrogen-sensitive PTPRO expression represses hepatocellular carcinoma progression by control of STAT3. Hepatology (2013) 57(2):678–88. doi: 10.1002/hep.25980 22821478

[141] Dai B Geng L Yu Y Sui C Xie F Shen W. Methylation patterns of estrogen receptor α promoter correlate with estrogen receptor α expression and clinicopathological factors in hepatocellular carcinoma. Exp Biol Med (Maywood) (2014) 239(7):883–90. doi: 10.1177/1535370214536651 24939822

[142] Chen JJ Tang YS Huang SF Ai JG Wang HX Zhang LP. HBx protein-induced upregulation of microRNA-221 promotes aberrant proliferation in HBV−related hepatocellular carcinoma by targeting estrogen receptor-α. Oncol Rep (2015) 33(2):792–8. doi: 10.3892/or.2014.3647 25483016

[143] Iyer JK Kalra M Kaul A Payton ME Kaul R. Estrogen receptor expression in chronic hepatitis c and hepatocellular carcinoma pathogenesis. World J Gastroenterol (2017) 23(37):6802–16. doi: 10.3748/wjg.v23.i37.6802 PMC5645614 29085224

[144] Yang W Lu Y Xu Y Xu L Zheng W Wu Y. Estrogen represses hepatocellular carcinoma (HCC) growth *via* inhibiting alternative activation of tumor-associated macrophages (TAMs). J Biol Chem (2012) 287(48):40140–9. doi: 10.1074/jbc.M112.348763 PMC3504728 22908233

[145] Lin YM Velmurugan BK Yeh YL Tu CC Ho TJ Lai TY. Activation of estrogen receptors with E2 downregulates peroxisome proliferator-activated receptor γ in hepatocellular carcinoma. Oncol Rep (2013) 30(6):3027–31. doi: 10.3892/or.2013.2793 24126791

[146] Wei T Chen W Wen L Zhang J Zhang Q Yang J. G Protein-coupled estrogen receptor deficiency accelerates liver tumorigenesis by enhancing inflammation and fibrosis. Cancer Lett (2016) 382(2):195–202. doi: 10.1016/j.canlet.2016.08.012 27594673

[147] Li Z Chen L Chu H Wang W Yang L. Estrogen alleviates hepatocyte necroptosis depending on GPER in hepatic ischemia reperfusion injury. J Physiol Biochem (2021) 78(1):125–37. doi: 10.1007/s13105-021-00846-5 34651286

[148] Liu SL Wu XS Li FN Yao WY Wu ZY Dong P. ERRα promotes pancreatic cancer progression by enhancing the transcription of PAI1 and activating the MEK/ERK pathway. Am J Cancer Res (2020) 10(11):3622–43. PMC7716152 33294258

[149] Pozios I Seel NN Hering NA Hartmann L Liu V Camaj P. Raloxifene inhibits pancreatic adenocarcinoma growth by interfering with ERβ and IL-6/gp130/STAT3 signaling. Cell Oncol (Dordr) (2021) 44(1):167–77. doi: 10.1007/s13402-020-00559-9 PMC7906944 32940862

[150] Gupta VK Banerjee S Saluja AK. Learning from gender disparity: Role of estrogen receptor activation in coping with pancreatic cancer. Cell Mol Gastroenterol Hepatol (2020) 10(4):862–3. doi: 10.1016/j.jcmgh.2020.07.009 PMC7573666 32798451

[151] Natale CA Li J Pitarresi JR Norgard RJ Dentchev T Capell BC. Pharmacologic activation of the G protein-coupled estrogen receptor inhibits pancreatic ductal adenocarcinoma. Cell Mol Gastroenterol Hepatol (2020) 10(4):868–80.e1. doi: 10.1016/j.jcmgh.2020.04.016 32376419 PMC7578406

[152] Prins GS Marmer M Woodham C Chang W Kuiper G Gustafsson JA. Estrogen receptor-beta messenger ribonucleic acid ontogeny in the prostate of normal and neonatally estrogenized rats. Endocrinology (1998) 139(3):874–83. doi: 10.1210/endo.139.3.5827 9492016

[153] Kauffman EC Robinson BD Downes M Marcinkiewicz K Vourganti S Scherr DS. Estrogen receptor-β expression and pharmacological targeting in bladder cancer. Oncol Rep (2013) 30(1):131–8. doi: 10.3892/or.2013.2416 PMC3729232 23612777

[154] Teng J Wang ZY Prossnitz ER Bjorling DE. The G protein-coupled receptor GPR30 inhibits human urothelial cell proliferation. Endocrinology (2008) 149(8):4024–34. doi: 10.1210/en.2007-1669 PMC2488207 18467434

[155] Foster TC. Role of estrogen receptor alpha and beta expression and signaling on cognitive function during aging. Hippocampus (2012) 22(4):656–69. doi: 10.1002/hipo.20935 PMC3704216 21538657

[156] Ishunina TA Swaab DF. Hippocampal estrogen receptor-alpha splice variant TADDI in the human brain in aging and alzheimer's disease. Neuroendocrinology (2009) 89(2):187–99. doi: 10.1159/000158573 18815440

[157] Lai Y-J Yu D Zhang JH Chen GJ. Cooperation of genomic and rapid nongenomic actions of estrogens in synaptic plasticity. Mol Neurobiol (2017) 54(6):4113–26. doi: 10.1007/s12035-016-9979-y PMC5509832 27324789

[158] Chhibber A Woody SK Karim Rumi MA Soares MJ Zhao L. Estrogen receptor β deficiency impairs BDNF-5-HT(2A) signaling in the hippocampus of female brain: A possible mechanism for menopausal depression. Psychoneuroendocrinology (2017) 82:107–16. doi: 10.1016/j.psyneuen.2017.05.016 PMC5523821 28544903

[159] Lymer J Robinson A Winters BD Choleris E. Rapid effects of dorsal hippocampal G-protein coupled estrogen receptor on learning in female mice. Psychoneuroendocrinology (2017) 77:131–40. doi: 10.1016/j.psyneuen.2016.11.019 28033587

[160] Wang Z-F Pan Z-Y Xu C-S Li Z-Q. Activation of G-protein coupled estrogen receptor 1 improves early-onset cognitive impairment *via* PI3K/Akt pathway in rats with traumatic brain injury. Biochem Biophys Res Commun (2017) 482(4):948–53. doi: 10.1016/j.bbrc.2016.11.138 27908726

[161] Bessa A Campos FL Videira RA Mendes-Oliveira J Bessa-Neto D Baltazar G. GPER: A new tool to protect dopaminergic neurons? Biochim Biophys Acta (BBA) - Mol Basis Dis (2015) 1852(10, Part A):2035–41. doi: 10.1016/j.bbadis.2015.07.004 26170064

[162] Tian W Pang W Ge Y He X Wang D Li X. Hepatocyte-generated 27-hydroxycholesterol promotes the growth of melanoma by activation of estrogen receptor alpha. J Cell Biochem (2018) 119(3):2929–38. doi: 10.1002/jcb.26498 29130512

[163] Marzagalli M Montagnani Marelli M Casati L Fontana F Moretti RM Limonta P. Estrogen receptor β in melanoma: From molecular insights to potential clinical utility. Front Endocrinol (Lausanne) (2016) 7:140. doi: 10.3389/fendo.2016.00140 27833586 PMC5080294

[164] Fábián M Rencz F Krenács T Brodszky V Hársing J Németh K. Expression of G protein-coupled oestrogen receptor in melanoma and in pregnancy-associated melanoma. J Eur Acad Dermatol Venereol (2017) 31(9):1453–61. doi: 10.1111/jdv.14304 28467693

[165] Natale CA Li J Zhang J Dahal A Dentchev T Stanger BZ. Activation of G protein-coupled estrogen receptor signaling inhibits melanoma and improves response to immune checkpoint blockade. Elife (2018) 7:e31770–89. doi: 10.7554/eLife.31770 29336307 PMC5770157

[166] Mińko A Turoń-Skrzypińska A Rył A Bargiel P Hilicka Z Michalczyk K. Endometriosis-a multifaceted problem of a modern woman. Int J Environ Res Public Health (2021) 18(15):8177–30. doi: 10.3390/ijerph18158177 34360470 PMC8346111

[167] Chantalat E Valera MC Vaysse C Noirrit E Rusidze M Weyl A. Estrogen receptors and endometriosis. Int J Mol Sci (2020) 21(8):2815–32. doi: 10.3390/ijms21082815 PMC7215544 32316608

[168] Enmark E Pelto-Huikko M Grandien K Lagercrantz S Lagercrantz J Fried G. Human estrogen receptor beta-gene structure, chromosomal localization, and expression pattern. J Clin Endocrinol Metab (1997) 82(12):4258–65. doi: 10.1210/jcem.82.12.4470 9398750

[169] Pellegrini C Gori I Achtari C Hornung D Chardonnens E Wunder D. The expression of estrogen receptors as well as GREB1, c-MYC, and cyclin D1, estrogen-regulated genes implicated in proliferation, is increased in peritoneal endometriosis. Fertil Steril (2012) 98(5):1200–8. doi: 10.1016/j.fertnstert.2012.06.056 22884659

[170] Samartzis N Samartzis EP Noske A Fedier A Dedes KJ Caduff R. Expression of estrogen receptor alpha and beta in peritoneal and ovarian endometriosis. Fertil Steril (2001) 75(6):1198–205. doi: 10.1016/S0015-0282(01)01783-6 11384649

[171] Qiu JJ Ye LC Ding JX Feng WW Jin HY Zhang Y. Expression and clinical significance of estrogen-regulated long non-coding RNAs in estrogen receptor α-positive ovarian cancer progression. Oncol Rep (2014) 31(4):1613–22. doi: 10.3892/or.2014.3000 24481591

[172] Cairns J Ingle JN Kalari KR Shepherd LE Kubo M Goetz MP. The lncRNA MIR2052HG regulates ERα levels and aromatase inhibitor resistance through LMTK3 by recruiting EGR1. Breast Cancer Res (2019) 21(1):47. doi: 10.1186/s13058-019-1130-3 30944027 PMC6448248

[173] Cheng CW Licence D Cook E Luo F Arends MJ Smith SK. Activation of mutated K-ras in donor endometrial epithelium and stroma promotes lesion growth in an intact immunocompetent murine model of endometriosis. J Pathol (2011) 224(2):261–9. doi: 10.1002/path.2852 21480232

[174] Xue Q Lin Z Cheng YH Huang CC Marsh E Yin P. Promoter methylation regulates estrogen receptor 2 in human endometrium and endometriosis. Biol Reprod (2007) 77(4):681–7. doi: 10.1095/biolreprod.107.061804 17625110

[175] Yotova I Hsu E Do C Gaba A Sczabolcs M Dekan S. Epigenetic alterations affecting transcription factors and signaling pathways in stromal cells of endometriosis. PloS One (2017) 12(1):e0170859. doi: 10.1371/journal.pone.0170859 28125717 PMC5268815

[176] Heublein S Vrekoussis T Kuhn C Friese K Makrigiannakis A Mayr D. Inducers of G-protein coupled estrogen receptor (GPER) in endometriosis: potential implications for macrophages and follicle maturation. J Reprod Immunol (2013) 97(1):95–103. doi: 10.1016/j.jri.2012.10.013 23432876

[177] Samartzis N Samartzis EP Noske A Fedier A Dedes KJ Caduff R. Expression of the G protein-coupled estrogen receptor (GPER) in endometriosis: a tissue microarray study. Reprod Biol Endocrinol (2012) 10:30. doi: 10.1186/1477-7827-10-30 22520060 PMC3443027

[178] Suzuki K Inaba S Takeuchi H Takezawa Y Fukabori Y Suzuki T. Endocrine environment of benign prostatic hyperplasia–relationships of sex steroid hormone levels with age and the size of the prostate. Nihon Hinyokika Gakkai Zasshi (1992) 83(5):664–71. doi: 10.5980/jpnjurol1989.83.664 1379658

[179] Gupta L Thakur H Sobti RC Seth A Singh SK. Role of genetic polymorphism of estrogen receptor-alpha gene and risk of prostate cancer in north Indian population. Mol Cell Biochem (2010) 335(1-2):255–61. doi: 10.1007/s11010-009-0275-2 19904497

[180] Di Zazzo E Galasso G Giovannelli P Di Donato M Di Santi A Cernera G. Prostate cancer stem cells: the role of androgen and estrogen receptors. Oncotarget (2016) 7(1):193–208. doi: 10.18632/oncotarget.6220 PMC4807992 26506594

[181] Lau KM To KF. Importance of estrogenic signaling and its mediated receptors in prostate cancer. Int J Mol Sci (2016) 17(9):1434–59. doi: 10.3390/ijms17091434 PMC5037713 27589731

[182] Ricke WA McPherson SJ Bianco JJ Cunha GR Wang Y Risbridger GP. Prostatic hormonal carcinogenesis is mediated by *in situ* estrogen production and estrogen receptor alpha signaling. FASEB J (2008) 22(5):1512–20. doi: 10.1096/fj.07-9526com 18055862

[183] Bonkhoff H Fixemer T Hunsicker I Remberger K. Estrogen receptor expression in prostate cancer and premalignant prostatic lesions. Am J Pathol (1999) 155(2):641–7. doi: 10.1016/S0002-9440(10)65160-7 PMC1866870 10433957

[184] Singh PB Matanhelia SS Martin FL. A potential paradox in prostate adenocarcinoma progression: oestrogen as the initiating driver. Eur J Cancer (2008) 44(7):928–36. doi: 10.1016/j.ejca.2008.02.051 18381236

[185] Fixemer T Remberger K Bonkhoff H. Differential expression of the estrogen receptor beta (ERbeta) in human prostate tissue, premalignant changes, and in primary, metastatic, and recurrent prostatic adenocarcinoma. Prostate (2003) 54(2):79–87. doi: 10.1002/pros.10171 12497580

[186] Leav I Lau KM Adams JY McNeal JE Taplin ME Wang J. Comparative studies of the estrogen receptors beta and alpha and the androgen receptor in normal human prostate glands, dysplasia, and in primary and metastatic carcinoma. Am J Pathol (2001) 159(1):79–92. doi: 10.1016/S0002-9440(10)61676-8 11438457 PMC1850428

[187] Qu LG Wardan H Davis ID Iddawela M Sluka P Pezaro CJ. Circulating oestrogen receptor mutations and splice variants in advanced prostate cancer. BJU Int (2019) 124 Suppl 1:50–6. doi: 10.1111/bju.14797 31090242

[188] Jilka RL Hangoc G Girasole G Passeri G Williams DC Abrams JS. Increased osteoclast development after estrogen loss: Mediation by interleukin-6. Science (1992) 257(5066):88–91. doi: 10.1126/science.1621100 1621100

[189] Pantschenko AG Zhang W Nahounou M McCarthy MB Stover ML Lichtler AC. Effect of osteoblast-targeted expression of bcl-2 in bone: differential response in male and female mice. J Bone Miner Res (2005) 20(8):1414–29. doi: 10.1359/JBMR.050315 16007339

[190] Cauley JA. Estrogen and bone health in men and women. Steroids (2015) 99:11–5. doi: 10.1016/j.steroids.2014.12.010 25555470

[191] Härkönen PL Väänänen HK. Monocyte-macrophage system as a target for estrogen and selective estrogen receptor modulators. Ann N Y Acad Sci (2006) 1089:218–27. doi: 10.1196/annals.1386.045 17261769

[192] Pacifici R. Estrogen deficiency, T cells and bone loss. Cell Immunol (2008) 252(1-2):68–80. doi: 10.1016/j.cellimm.2007.06.008 17888417

[193] de Giorgi V Mavilia C Massi D Gozzini A Aragona P Tanini A. Estrogen receptor expression in cutaneous melanoma: a real-time reverse transcriptase-polymerase chain reaction and immunohistochemical study. Arch Dermatol (2009) 145(1):30–6. doi: 10.1001/archdermatol.2008.537 19153340

[194] Lee KC Jessop H Suswillo R Zaman G Lanyon LE. The adaptive response of bone to mechanical loading in female transgenic mice is deficient in the absence of oestrogen receptor-alpha and -beta. J Endocrinol (2004) 182(2):193–201. doi: 10.1677/joe.0.1820193 15283680

[195] Kang WB Deng YT Wang DS Feng D Liu Q Wang XS. Osteoprotective effects of estrogen membrane receptor GPR30 in ovariectomized rats. J Steroid Biochem Mol Biol (2015) 154:237–44. doi: 10.1016/j.jsbmb.2015.07.002 26187146

[196] Iravani M Lagerquist MK Karimian E Chagin AS Ohlsson C Sävendahl L. Effects of the selective GPER1 agonist G1 on bone growth. Endocr Connect (2019) 8(9):1302–9. doi: 10.1530/EC-19-0274 PMC6765336 31434056

[197] Siegfried JM. Women and lung cancer: does oestrogen play a role? Lancet Oncol (2001) 2(8):506–13. doi: 10.1016/S1470-2045(01)00457-0 11905727

[198] Landis SH Murray T Bolden S Wingo PA. Cancer statistics, 1999. CA Cancer J Clin (1999) 49(1):8–31, 1. doi: 10.3322/canjclin.49.1.8 10200775

[199] Hsu LH Liu KJ Tsai MF Wu CR Feng AC Chu NM. Estrogen adversely affects the prognosis of patients with lung adenocarcinoma. Cancer Sci (2015) 106(1):51–9. doi: 10.1111/cas.12558 PMC4317775 25338663

[200] Mollerup S Jørgensen K Berge G Haugen A. Expression of estrogen receptors alpha and beta in human lung tissue and cell lines. Lung Cancer (2002) 37(2):153–9. doi: 10.1016/S0169-5002(02)00039-9 12140138

[201] Kawai H Ishii A Washiya K Konno T Kon H Yamaya C. Estrogen receptor alpha and beta are prognostic factors in non-small cell lung cancer. Clin Cancer Res (2005) 11(14):5084–9. doi: 10.1158/1078-0432.CCR-05-0200 16033821

[202] Carey MA Card JW Voltz JW Germolec DR Korach KS Zeldin DC. The impact of sex and sex hormones on lung physiology and disease: lessons from animal studies. Am J Physiol Lung Cell Mol Physiol (2007) 293(2):L272–8. doi: 10.1152/ajplung.00174.2007 17575008

[203] Brandenberger AW Tee MK Lee JY Chao V Jaffe RB. Tissue distribution of estrogen receptors alpha (ER-alpha) and beta (ER-beta) mRNA in the midgestational human fetus. J Clin Endocrinol Metab (1997) 82(10):3509–12. doi: 10.1210/jcem.82.10.4400 9329394

[204] Morani A Barros RP Imamov O Hultenby K Arner A Warner M. Lung dysfunction causes systemic hypoxia in estrogen receptor beta knockout (ERbeta-/-) mice. Proc Natl Acad Sci U.S.A. (2006) 103(18):7165–9. doi: 10.1073/pnas.0602194103 PMC1459034 16636272

[205] Omoto Y Kobayashi Y Nishida K Tsuchiya E Eguchi H Nakagawa K. Expression, function, and clinical implications of the estrogen receptor beta in human lung cancers. Biochem Biophys Res Commun (2001) 285(2):340–7. doi: 10.1006/bbrc.2001.5158 11444848

[206] Baik CS Eaton KD. Estrogen signaling in lung cancer: an opportunity for novel therapy. Cancers (Basel) (2012) 4(4):969–88. doi: 10.3390/cancers4040969 PMC3712734 24213497

[207] Kawai H. Estrogen receptors as the novel therapeutic biomarker in non-small cell lung cancer. World J Clin Oncol (2014) 5(5):1020–7. doi: 10.5306/wjco.v5.i5.1020 PMC4259928 25493237

[208] Leung YK Mak P Hassan S Ho SM. Estrogen receptor (ER)-beta isoforms: a key to understanding ER-beta signaling. Proc Natl Acad Sci U.S.A. (2006) 103(35):13162–7. doi: 10.1073/pnas.0605676103 PMC1552044 16938840

[209] Bai Y Shen W Zhang L Yang Z Xiong L Tang H. Oestrogen receptor β5 and epidermal growth factor receptor synergistically promote lung cancer progression. Autoimmunity (2018) 51(4):157–65. doi: 10.1080/08916934.2018.1486825 30022688

[210] Jala VR Radde BN Haribabu B Klinge CM. Enhanced expression of G-protein coupled estrogen receptor (GPER/GPR30) in lung cancer. BMC Cancer (2012) 12:624. doi: 10.1186/1471-2407-12-624 23273253 PMC3557142

[211] Słowikowski BK Lianeri M Jagodziński PP. Exploring estrogenic activity in lung cancer. Mol Biol Rep (2017) 44(1):35–50. doi: 10.1007/s11033-016-4086-8 27783191 PMC5310573

[212] Kurt AH Çelik A Kelleci BM. Oxidative/antioxidative enzyme-mediated antiproliferative and proapoptotic effects of the GPER1 agonist G-1 on lung cancer cells. Oncol Lett (2015) 10(5):3177–82. doi: 10.3892/ol.2015.3711 PMC4665271 26722308

[213] Iorga A Cunningham CM Moazeni S Ruffenach G Umar S Eghbali M. The protective role of estrogen and estrogen receptors in cardiovascular disease and the controversial use of estrogen therapy. Biol Sex Differ (2017) 8(1):33. doi: 10.1186/s13293-017-0152-8 29065927 PMC5655818

[214] Savarese G Lund LH. Global public health burden of heart failure. Card Fail Rev (2017) 3(1):7–11. doi: 10.15420/cfr.2016:25:2 28785469 PMC5494150

[215] Hayward CS Kelly RP Collins P. The roles of gender, the menopause and hormone replacement on cardiovascular function. Cardiovasc Res (2000) 46(1):28–49. doi: 10.1016/S0008-6363(00)00005-5 10727651

[216] Aryan L Younessi D Zargari M Banerjee S Agopian J Rahman S. The role of estrogen receptors in cardiovascular disease. Int J Mol Sci (2020) 21(12):4314. doi: 10.3390/ijms21124314 PMC7352426 32560398

[217] Wang M Crisostomo P Wairiuko GM Meldrum DR. Estrogen receptor-alpha mediates acute myocardial protection in females. Am J Physiol Heart Circ Physiol (2006) 290(6):H2204–9. doi: 10.1152/ajpheart.01219.2005 16415070

[218] Hamada H Kim MK Iwakura A Ii M Thorne T Qin G. Estrogen receptors alpha and beta mediate contribution of bone marrow-derived endothelial progenitor cells to functional recovery after myocardial infarction. Circulation (2006) 114(21):2261–70. doi: 10.1161/CIRCULATIONAHA.106.631465 17088460

[219] Lyle AN Griendling KK. Modulation of vascular smooth muscle signaling by reactive oxygen species. Physiol (Bethesda) (2006) 21:269–80. doi: 10.1152/physiol.00004.2006 16868316

[220] Zhu L Shi J Luu TN Neuman JC Trefts E Yu S. Hepatocyte estrogen receptor alpha mediates estrogen action to promote reverse cholesterol transport during Western-type diet feeding. Mol Metab (2018) 8:106–16. doi: 10.1016/j.molmet.2017.12.012 PMC5985047 29331506

[221] Mendelsohn ME. Protective effects of estrogen on the cardiovascular system. Am J Cardiol (2002) 89(12a):12E–17E; discussion 17E-18E. doi: 10.1016/S0002-9149(02)02405-0 12084397

[222] McRobb LS Shi J Luu TN Neuman JC Trefts E Yu S. Estrogen receptor control of atherosclerotic calcification and smooth muscle cell osteogenic differentiation. Arterioscler Thromb Vasc Biol (2017) 37(6):1127–37. doi: 10.1161/ATVBAHA.117.309054 28473445

[223] Owens GK Kumar MS Wamhoff BR. Molecular regulation of vascular smooth muscle cell differentiation in development and disease. Physiol Rev (2004) 84(3):767–801. doi: 10.1152/physrev.00041.2003 15269336

[224] Smirnova NF Fontaine C Buscato M Lupieri A Vinel A Valera MC. The activation function-1 of estrogen receptor alpha prevents arterial neointima development through a direct effect on smooth muscle cells. Circ Res (2015) 117(9):770–8. doi: 10.1161/CIRCRESAHA.115.306416 PMC4596486 26316608

[225] Menazza S Murphy E. The expanding complexity of estrogen receptor signaling in the cardiovascular system. Circ Res (2016) 118(6):994–1007. doi: 10.1161/CIRCRESAHA.115.305376 26838792 PMC5012719

[226] Fredette NC Meyer MR Prossnitz ER. Role of GPER in estrogen-dependent nitric oxide formation and vasodilation. J Steroid Biochem Mol Biol (2018) 176:65–72. doi: 10.1016/j.jsbmb.2017.05.006 28529128 PMC5694388

[227] Chen C Gong X Yang X Shang X Du Q Liao Q. The roles of estrogen and estrogen receptors in gastrointestinal disease. Oncol Lett (2019) 18(6):5673–80. doi: 10.3892/ol.2019.10983 PMC6865762 31788039

[228] Kurt D Saruhan BG Kanay Z Yokus B Kanay BE Unver O. Effect of ovariectomy and female sex hormones administration upon gastric ulceration induced by cold and immobility restraint stress. Saudi Med J (2007) 28(7):1021–7. 17603703

[229] Sangma TK Jain S Mediratta PK. Effect of ovarian sex hormones on non-steroidal anti-inflammatory drug-induced gastric lesions in female rats. Indian J Pharmacol (2014) 46(1):113–6. doi: 10.4103/0253-7613.125191 PMC3912794 24550596

[230] Meleine M Matricon J. Gender-related differences in irritable bowel syndrome: potential mechanisms of sex hormones. World J Gastroenterol (2014) 20(22):6725–43. doi: 10.3748/wjg.v20.i22.6725 PMC4051914 24944465

[231] Bustos V Nolan ÁM Nijhuis A Harvey H Parker A Poulsom R. GPER mediates differential effects of estrogen on colon cancer cell proliferation and migration under normoxic and hypoxic conditions. Oncotarget (2017) 8(48):84258–75. doi: 10.18632/oncotarget.20653 PMC5663593 29137421

[232] Iijima K Shimosegawa T. Involvement of luminal nitric oxide in the pathogenesis of the gastroesophageal reflux disease spectrum. J Gastroenterol Hepatol (2014) 29(5):898–905. doi: 10.1111/jgh.12548 24863184

[233] Vizcaino AP Moreno V Lambert R Parkin DM. Time trends incidence of both major histologic types of esophageal carcinomas in selected countries, 1973-1995. Int J Cancer (2002) 99(6):860–8. doi: 10.1002/ijc.10427 12115489

[234] Boeckxstaens G El-Serag HB Smout AJ Kahrilas PJ. Symptomatic reflux disease: the present, the past and the future. Gut (2014) 63(7):1185–93. doi: 10.1136/gutjnl-2013-306393 PMC4078752 24607936

[235] Wang BJ Zhang B Yan SS Li ZC Jiang T Hua CJ. Hormonal and reproductive factors and risk of esophageal cancer in women: a meta-analysis. Dis Esophagus (2016) 29(5):448–54. doi: 10.1111/dote.12349 25809699

[236] Wang QM Yuan L Qi YJ Ma ZY Wang LD. Estrogen analogues: promising target for prevention and treatment of esophageal squamous cell carcinoma in high risk areas. Med Sci Monit (2010) 16(7):Hy19–22. 20581783

[237] Nilsson M Johnsen R Ye W Hveem K Lagergren J. Obesity and estrogen as risk factors for gastroesophageal reflux symptoms. Jama (2003) 290(1):66–72. doi: 10.1001/jama.290.1.66 12837713

[238] Okada K Inamori M Imajyo K Chiba H Nonaka T Shiba T. Gender differences of low-dose aspirin-associated gastroduodenal ulcer in Japanese patients. World J Gastroenterol (2010) 16(15):1896–900. doi: 10.3748/wjg.v16.i15.1896 PMC2856832 20397269

[239] Speir E Yu ZX Takeda K Ferrans VJ Cannon RO 3rd. Antioxidant effect of estrogen on cytomegalovirus-induced gene expression in coronary artery smooth muscle cells. Circulation (2000) 102(24):2990–6. doi: 10.1161/01.CIR.102.24.2990 11113051

[240] Chung HW Noh SH Lim JB. Analysis of demographic characteristics in 3242 young age gastric cancer patients in Korea. World J Gastroenterol (2010) 16(2):256–63. doi: 10.3748/wjg.v16.i2.256 PMC2806566 20066747

[241] Tokunaga A Kojima N Andoh T Matsukura N Yoshiyasu M Tanaka N. Hormone receptors in gastric cancer. Eur J Cancer Clin Oncol (1983) 19(5):687–9. doi: 10.1016/0277-5379(83)90186-4 6683640

[242] Furukawa H Iwanaga T Koyama H Taniguchi H. Effect of sex hormones on the experimental induction of cancer in rat stomach - a preliminary study. Digestion (1982) 23(3):151–5. doi: 10.1159/000198722 7106416

[243] Wang M Pan JY Song GR Chen HB An LJ Qu SX. Altered expression of estrogen receptor alpha and beta in advanced gastric adenocarcinoma: correlation with prothymosin alpha and clinicopathological parameters. Eur J Surg Oncol (2007) 33(2):195–201. doi: 10.1016/j.ejso.2006.09.009 17046193

[244] Takano N Iizuka N Hazama S Yoshino S Tangoku A Oka M. Expression of estrogen receptor-alpha and -beta mRNAs in human gastric cancer. Cancer Lett (2002) 176(2):129–35. doi: 10.1016/S0304-3835(01)00739-X 11804739

[245] Qin J Liu M Ding Q Ji X Hao Y Wu X. The direct effect of estrogen on cell viability and apoptosis in human gastric cancer cells. Mol Cell Biochem (2014) 395(1-2):99–107. doi: 10.1007/s11010-014-2115-2 24934239

[246] Fu Z Wang X Zhou H Li Y Chen Y Wang Z. GRP78 positively regulates estrogen−stimulated cell growth mediated by ER−α36 in gastric cancer cells. Mol Med Rep (2017) 16(6):8329–34. doi: 10.3892/mmr.2017.7615 28983626

[247] Fu Z Deng H Wang X Yang X Wang Z Liu L. Involvement of ER-α36 in the malignant growth of gastric carcinoma cells is associated with GRP94 overexpression. Histopathology (2013) 63(3):325–33. doi: 10.1111/his.12171 23829397

[248] Heitkemper MM Chang L. Do fluctuations in ovarian hormones affect gastrointestinal symptoms in women with irritable bowel syndrome? Gender Med (2009) 6 Suppl 2(Suppl 2):152–67. doi: 10.1016/j.genm.2009.03.004 PMC3322543 19406367

[249] Cook LC Hillhouse AE Myles MH Lubahn DB Bryda EC Davis JW. The role of estrogen signaling in a mouse model of inflammatory bowel disease: a helicobacter hepaticus model. PloS One (2014) 9(4):e94209. doi: 10.1371/journal.pone.0094209 24709804 PMC3978010

[250] Principi M Barone M Pricci M De Tullio N Losurdo G Ierardi E. Ulcerative colitis: from inflammation to cancer. do estrogen receptors have a role? World J Gastroenterol (2014) 20(33):11496–504. doi: 10.3748/wjg.v20.i33.11496 PMC4155343 25206257

[251] Looijer-van Langen M Hotte N Dieleman LA Albert E Mulder C Madsen KL. Estrogen receptor-β signaling modulates epithelial barrier function. Am J Physiol Gastrointest Liver Physiol (2011) 300(4):G621–6. doi: 10.1152/ajpgi.00274.2010 21252046

[252] Jiang H Teng R Wang Q Zhang X Wang H Wang Z. Transcriptional analysis of estrogen receptor alpha variant mRNAs in colorectal cancers and their matched normal colorectal tissues. J Steroid Biochem Mol Biol (2008) 112(1-3):20–4. doi: 10.1016/j.jsbmb.2008.07.004 18703141

[253] Campbell-Thompson M Lynch IJ Bhardwaj B. Expression of estrogen receptor (ER) subtypes and ERbeta isoforms in colon cancer. Cancer Res (2001) 61(2):632–40. 11212261

[254] Wong NA Malcomson RD Jodrell DI Groome NP Harrison DJ Saunders PT. ERbeta isoform expression in colorectal carcinoma: an *in vivo* and *in vitro* study of clinicopathological and molecular correlates. J Pathol (2005) 207(1):53–60. doi: 10.1002/path.1807 15954165

[255] Wang AG Lee KY Kim SY Choi JY Lee KH Kim WH. The expression of estrogen receptors in hepatocellular carcinoma in Korean patients. Yonsei Med J (2006) 47(6):811–6. doi: 10.3349/ymj.2006.47.6.811 PMC2687821 17191310

[256] De Maria N Manno M Villa E. Sex hormones and liver cancer. Mol Cell Endocrinol (2002) 193(1-2):59–63. doi: 10.1016/S0303-7207(02)00096-5 12161002

[257] Ezhilarasan D. Critical role of estrogen in the progression of chronic liver diseases. Hepatobil Pancreat Dis Int (2020) 19(5):429–34. doi: 10.1016/j.hbpd.2020.03.011 32299655

[258] Baldissera VD Alves AF Almeida S Porawski M Giovenardi M. Hepatocellular carcinoma and estrogen receptors: Polymorphisms and isoforms relations and implications. Med Hypotheses (2016) 86:67–70. doi: 10.1016/j.mehy.2015.11.030 26804600

[259] Kao TL Kuan YP Cheng WC Chang WC Jeng LB Yeh S. Estrogen receptors orchestrate cell growth and differentiation to facilitate liver regeneration. Theranostics (2018) 8(10):2672–82. doi: 10.7150/thno.23624 PMC5957001 29774067

[260] Hishida M Nomoto S Inokawa Y Hayashi M Kanda M Okamura Y. Estrogen receptor 1 gene as a tumor suppressor gene in hepatocellular carcinoma detected by triple-combination array analysis. Int J Oncol (2013) 43(1):88–94. doi: 10.3892/ijo.2013.1951 23695389

[261] Li CL Yeh KH Liu WH Chen CL Chen DS Chen PJ. Elevated p53 promotes the processing of miR-18a to decrease estrogen receptor-α in female hepatocellular carcinoma. Int J Cancer (2015) 136(4):761–70. doi: 10.1002/ijc.29052 24975878

[262] Han SJ Xu GL Jia WD Wang YC Li JS Ma JL. [Expression of estrogen receptor α in hepatitis b-related hepatocellular carcinoma and its clinical significance]. Zhonghua Wai Ke Za Zhi (2010) 48(24):1875–80. 21211272

[263] Villa E Colantoni A Grottola A Ferretti I Buttafoco P Bertani H. Variant estrogen receptors and their role in liver disease. Mol Cell Endocrinol (2002) 193(1-2):65–9. doi: 10.1016/S0303-7207(02)00097-7 12161003

[264] Qin H Song Z Shaukat H Zheng W. Genistein regulates lipid metabolism *via* estrogen receptor β and its downstream signal Akt/mTOR in HepG2 cells. Nutrients (2021) 13(11):4015–29. doi: 10.3390/nu13114015 34836271 PMC8622023

[265] Lin L Zhou M Que R Chen Y Liu X Zhang K. Saikosaponin-d protects against liver fibrosis by regulating the estrogen receptor-β/NLRP3 inflammasome pathway. Biochem Cell Biol (2021) 99(5):666–74. doi: 10.1139/bcb-2020-0561 33974808

[266] Zhang B Zhang CG Ji LH Zhao G Wu ZY. Estrogen receptor β selective agonist ameliorates liver cirrhosis in rats by inhibiting the activation and proliferation of hepatic stellate cells. J Gastroenterol Hepatol (2018) 33(3):747–55. doi: 10.1111/jgh.13976 28884481

[267] Mauvais-Jarvis F Clegg DJ Hevener AL. The role of estrogens in control of energy balance and glucose homeostasis. Endocr Rev (2013) 34(3):309–38. doi: 10.1210/er.2012-1055 PMC3660717 23460719

[268] Satake M Sawai H Go VL Satake K Reber HA Hines OJ. Estrogen receptors in pancreatic tumors. Pancreas (2006) 33(2):119–27. doi: 10.1097/01.mpa.0000226893.09194.ec 16868476

[269] Estrella JS Ma LT Milton DR Yao JC Wang H Rashid A. Expression of estrogen-induced genes and estrogen receptor β in pancreatic neuroendocrine tumors: implications for targeted therapy. Pancreas (2014) 43(7):996–1002. doi: 10.1097/MPA.0000000000000203 25058880 PMC4628823

[270] Lykoudis PM Contis J. Estrogen receptor expression in pancreatic adenocarcinoma: Time to reconsider evidence. Pancreas (2021) 50(9):1250–3. doi: 10.1097/MPA.0000000000001921 34860807

[271] Sen A Iyer J Boddu S Kaul A Kaul R. Estrogen receptor alpha differentially modulates host immunity in the bladder and kidney in response to urinary tract infection. Am J Clin Exp Urol (2019) 7(3):110–22. PMC6627544 31317051

[272] Wang C Symington JW Ma E Cao B Mysorekar IU. Estrogenic modulation of uropathogenic escherichia coli infection pathogenesis in a murine menopause model. Infect Immun (2013) 81(3):733–9. doi: 10.1128/IAI.01234-12 PMC3584860 23264047

[273] Chen Y-Y Su T-H Lau H-H. Estrogen for the prevention of recurrent urinary tract infections in postmenopausal women: a meta-analysis of randomized controlled trials. Int Urogynecol J (2021) 32(1):17–25. doi: 10.1007/s00192-020-04397-z 32564121

[274] Dwyer PL O'Reilly M. Recurrent urinary tract infection in the female. Curr Opin Obstet Gynecol (2002) 14(5):537–43. doi: 10.1097/00001703-200210000-00016 12401984

[275] Stamm WE Raz R. Factors contributing to susceptibility of postmenopausal women to recurrent urinary tract infections. Clin Infect Dis (1999) 28(4):723–5. doi: 10.1086/515209 10825026

[276] Hextall A. Oestrogens and lower urinary tract function. Maturitas (2000) 36(2):83–92. doi: 10.1016/S0378-5122(00)00143-2 11006496

[277] Teng J Wang ZY Jarrard DF Bjorling DE. Roles of estrogen receptor alpha and beta in modulating urothelial cell proliferation. Endocr Relat Cancer (2008) 15(1):351–64. doi: 10.1677/erc.1.01255 PMC3513362 18310301

[278] Mäkelä S Strauss L Kuiper G Valve E Salmi S Santti R. Differential expression of estrogen receptors alpha and beta in adult rat accessory sex glands and lower urinary tract. Mol Cell Endocrinol (2000) 164(1-2):109–16. doi: 10.1016/S0303-7207(00)00233-1 11026563

[279] Kaufmann O Baume H Dietel M. Detection of oestrogen receptors in non-invasive and invasive transitional cell carcinomas of the urinary bladder using both conventional immunohistochemistry and the tyramide staining amplification (TSA) technique. J Pathol (1998) 186(2):165–8. doi: 10.1002/(SICI)1096-9896(1998100)186:2<165::AID-PATH155>3.0.CO;2-Y 9924432

[280] Dubois B Hampel H Feldman HH Scheltens P Aisen P Andrieu S. Preclinical alzheimer's disease: definition, natural history, and diagnostic criteria. Alzheimer's Dementia (2016) 12(3):292–323. doi: 10.1016/j.jalz.2016.02.002 PMC6417794 27012484

[281] Zagni E Simoni L Colombo D. Sex and gender differences in central nervous system-related disorders. Neurosci J (2016) 2016:2827090–103. doi: 10.1155/2016/2827090 27314003 PMC4904110

[282] Bustamante-Barrientos FA Méndez-Ruette M Ortloff A Luz-Crawford P Rivera FJ Figueroa CD. The impact of estrogen and estrogen-like molecules in neurogenesis and neurodegeneration: Beneficial or harmful? Front Cell Neurosci (2021) 15:636176. doi: 10.3389/fncel.2021.636176 33762910 PMC7984366

[283] Overk CR Lu PY Wang YT Choi J Shaw JW Thatcher GR. Effects of aromatase inhibition versus gonadectomy on hippocampal complex amyloid pathology in triple transgenic mice. Neurobiol Dis (2012) 45(1):479–87. doi: 10.1016/j.nbd.2011.08.035 PMC3225601 21945538

[284] Yaffe K Haan M Byers A Tangen C Kuller K. Estrogen use APOE and cognitive decline: evidence of gene-environment interaction. Neurology (2000) 54(10):1949–54. doi: 10.1212/WNL.54.10.1949 10822435

[285] Song YJ Li SR Li XW Chen X Wei ZX Liu QS. The effect of estrogen replacement therapy on alzheimer's disease and parkinson's disease in postmenopausal women: A meta-analysis. Front Neurosci (2020) 14:157. doi: 10.3389/fnins.2020.00157 32210745 PMC7076111

[286] Ragonese P D'Amelio M Salemi G Aridon P Gammino M Epifanio A. Risk of Parkinson disease in women: effect of reproductive characteristics. Neurology (2004) 62(11):2010–4. doi: 10.1212/WNL.62.11.2010 15184606

[287] Al Sweidi S Sánchez MG Bourque M Morissette M Dluzen D Di Paolo T. Oestrogen receptors and signalling pathways: implications for neuroprotective effects of sex steroids in parkinson's disease. J Neuroendocrinol (2012) 24(1):48–61. doi: 10.1111/j.1365-2826.2011.02193.x 21790809

[288] Shulman LM. Is there a connection between estrogen and parkinson's disease? Parkinsonism Relat Disord (2002) 8(5):289–95. doi: 10.1016/S1353-8020(02)00014-7 15177058

[289] Lan YL Zhao J Li S. Update on the neuroprotective effect of estrogen receptor alpha against alzheimer's disease. J Alzheimers Dis (2015) 43(4):1137–48. doi: 10.3233/JAD-141875 25159676

[290] Yu Z Gao W Jiang E Lu F Zhang L Shi Z. Interaction between IGF-IR and ER induced by E2 and IGF-I. PloS One (2013) 8(5):e62642. doi: 10.1371/journal.pone.0062642 23704881 PMC3660452

[291] Zhao L Woody SK Chhibber A. Estrogen receptor β in alzheimer's disease: From mechanisms to therapeutics. Ageing Res Rev (2015) 24(Pt B):178–90. doi: 10.1016/j.arr.2015.08.001 PMC4661108 26307455

[292] Roque C Mendes-Oliveira J Duarte-Chendo C Baltazar G. The role of G protein-coupled estrogen receptor 1 on neurological disorders. Front Neuroendocrinol (2019) 55:100786. doi: 10.1016/j.yfrne.2019.100786 31513775

[293] Dika E Patrizi A Lambertini M Manuelpillai N Fiorentino M Altimari A. Estrogen receptors and melanoma: A review. Cells (2019) 8(11):1463–76. doi: 10.3390/cells8111463 PMC6912660 31752344

[294] Joosse A Collette S Suciu S Nijsten T Lejeune F Kleeberg UR. Superior outcome of women with stage I/II cutaneous melanoma: Pooled analysis of four European organisation for research and treatment of cancer phase III trials. J Clin Oncol (2012) 30(18):2240–7. doi: 10.1200/JCO.2011.38.0584 22547594

[295] Mervic L Leiter U Meier F Eigentler T Forschner A Metzler G. Sex differences in survival of cutaneous melanoma are age dependent: an analysis of 7338 patients. Melanoma Res (2011) 21(3):244–52. doi: 10.1097/CMR.0b013e32834577c8 21540649

[296] Richardson B Price A Wagner M Williams V Lorigan P Browne S. Investigation of female survival benefit in metastatic melanoma. Br J Cancer (1999) 80(12):2025–33. doi: 10.1038/sj.bjc.6690637 PMC2363135 10471056

[297] Rajabi P Bagheri M Hani M. Expression of estrogen receptor alpha in malignant melanoma. Adv BioMed Res (2017) 6:14. doi: 10.4103/2277-9175.200789 28299306 PMC5343608

[298] Bhari N Schwaertz RA Apalla Z Salerni G Akay BN Patil A. Effect of estrogen in malignant melanoma. J Cosmet Dermatol (2022) 21(5):1905–12. doi: 10.1111/jocd.14391 34416066

[299] de Giorgi V Gori A Gandini S Papi F Grazzini M Rossari S. Oestrogen receptor beta and melanoma: a comparative study. Br J Dermatol (2013) 168(3):513–9. doi: 10.1111/bjd.12056 23013061

[300] Spyropoulos C Melachrinou M Vasilakos P Tzorakoleftherakis E. Expression of estrogen receptors in melanoma and sentinel lymph nodes; a "female" clinical entity or a possible treatment modality? Eur J Gynaecol Oncol (2015) 36(2):123–30. 26050347

[301] Zhou JH Kim KB Myers JN Fox PS Ning J Bassett RL. Immunohistochemical expression of hormone receptors in melanoma of pregnant women, nonpregnant women, and men. Am J Dermatopathol (2014) 36(1):74–9. doi: 10.1097/DAD.0b013e3182914c64 PMC3795893 23812018

[302] Ribeiro MPC Santos AE Custódio JBA. The activation of the G protein-coupled estrogen receptor (GPER) inhibits the proliferation of mouse melanoma K1735-M2 cells. Chem Biol Interact (2017) 277:176–84. doi: 10.1016/j.cbi.2017.09.017 28947257

